# Expression, characterization, and activity optimization of a novel cellulase from the thermophilic bacteria *Cohnella *sp. A01

**DOI:** 10.1038/s41598-022-14651-7

**Published:** 2022-06-18

**Authors:** Shima Mohammadi, Hossein Tarrahimofrad, Sareh Arjmand, Javad Zamani, Kamahldin Haghbeen, Saeed Aminzadeh

**Affiliations:** 1 Bioprocess Engineering Group, Institute of Industrial and Environmental Biotechnology, National Institute of Genetic Engineering and Biotechnology (NIGEB), Tehran, Iran; 2 Protein Research Center, Shahid Beheshti University, Tehran, Iran

**Keywords:** Biotechnology, Molecular biology

## Abstract

Cellulases are hydrolytic enzymes with wide scientific and industrial applications. We described a novel cellulase, CelC307, from the thermophilic indigenous *Cohnella *sp. A01. The 3-D structure of the CelC307 was predicted by comparative modeling. Docking of CelC307 with specific inhibitors and molecular dynamic (MD) simulation revealed that these ligands bound in a non-competitive manner. The CelC307 protein was purified and characterized after recombinant expression in *Escherichia coli* (*E. coli*) BL21. Using CMC 1% as the substrate, the thermodynamic values were determined as K_m_ 0.46 mM, k_cat_ 104.30 × 10^–3^ (S^−1^), and k_cat_/K_m_ 226.73 (M^−1^ S^−1^). The CelC307 was optimally active at 40 °C and pH 7.0. The culture condition was optimized for improved CelC307 expression using Plackett–Burman and Box–Behnken design as follows: temperature 20 °C, pH 7.5, and inoculation concentration with an OD_600_ = 1. The endoglucanase activity was positively modulated in the presence of Na^+^, Li^+^, Ca^2+^, 2-mercaptoethanol (2-ME), and glycerol. The thermodynamic parameters calculated for CelC307 confirmed its inherent thermostability. The characterized CelC307 may be a suitable candidate for various biotechnological applications.

## Introduction

The increasing global energy demand, coupled with the depletion of fossil reserves and frightening environmental effects in the context of climate change, has directed the scientific interests toward greener alternative energy resources^1^. Photosynthesis produces about 150–170 × 10^9^ tons of lignocellulosic biomass, the most abundant renewable biomaterial, annually. Bioconversion of this biomass can conquer the global energy crises in an eco-friendly way. Lignocellulosic wastes are mainly composed of 30–50% cellulose, 15–35% hemicellulose, and 10–20% lignin^2^. Cellulose is an organic polymer made from simple monosaccharides and further degraded into short polysaccharides, simple sugars, and then into biofuels by a set of enzymes called cellulase. Industrial production of these enzymes found a broad spectrum of applications in many fields. Moreover, the increasing interest in converting lignocellulosic biomass to fermentable sugars for the production of bioethanol, promising alternative petrol, has generated a growing request for cellulases and their related enzymes^3,4^. Depending on the mode of action and the substrate specificity, the cellulose hydrolyzing enzymes are allocated into three main classes; [I] Exoglucanase or cellobiohydrolases (CBH) (EC 3.2.1.91) that acts on the reducing or non-reducing ends of cellulases and cleaves the cellobiose from glucan chains, [II] Endoglucanase (EG) (EC 3.2.1.4) that randomly cut β-1,4-glycosidic bonds of the cellulose string, and providing more ‘ends’ for exoglucanase, and [III] β-glucosidase (EC 3.2.1.21) which resolve cellulose and short-chain oligosaccharides to liberate glucose^5^. Despite being found in different members of the animal and plant kingdoms, cellulases are produced chiefly by fungi, bacteria, and protozoans that decompose cellulosic materials^6^. The thermal stability of an enzyme is a crucial characteristic that is desired in many industrial processes. Thermostable enzymes can increase the reaction rate in thermal conditions, where higher temperatures leads to reduce the substrates viscosity and increase the solubility^7^. Production of industrial enzymes using their native host has its own limitations and difficulties. Recombinant DNA technology has been employed to overcome the drawbacks of the economical production and characterization of various enzymes in a large-scale and regulated manner^7^. This paper describes the expression and characterization of a newly isolated cellulase obtained from thermophilic indigenous *Cohnella *sp. A01, as well as optimization of the enzyme activity.

## Results

### In silico structural characterization, phylogenetic analysis, and homology modeling of CelC307

**Figure 1:**
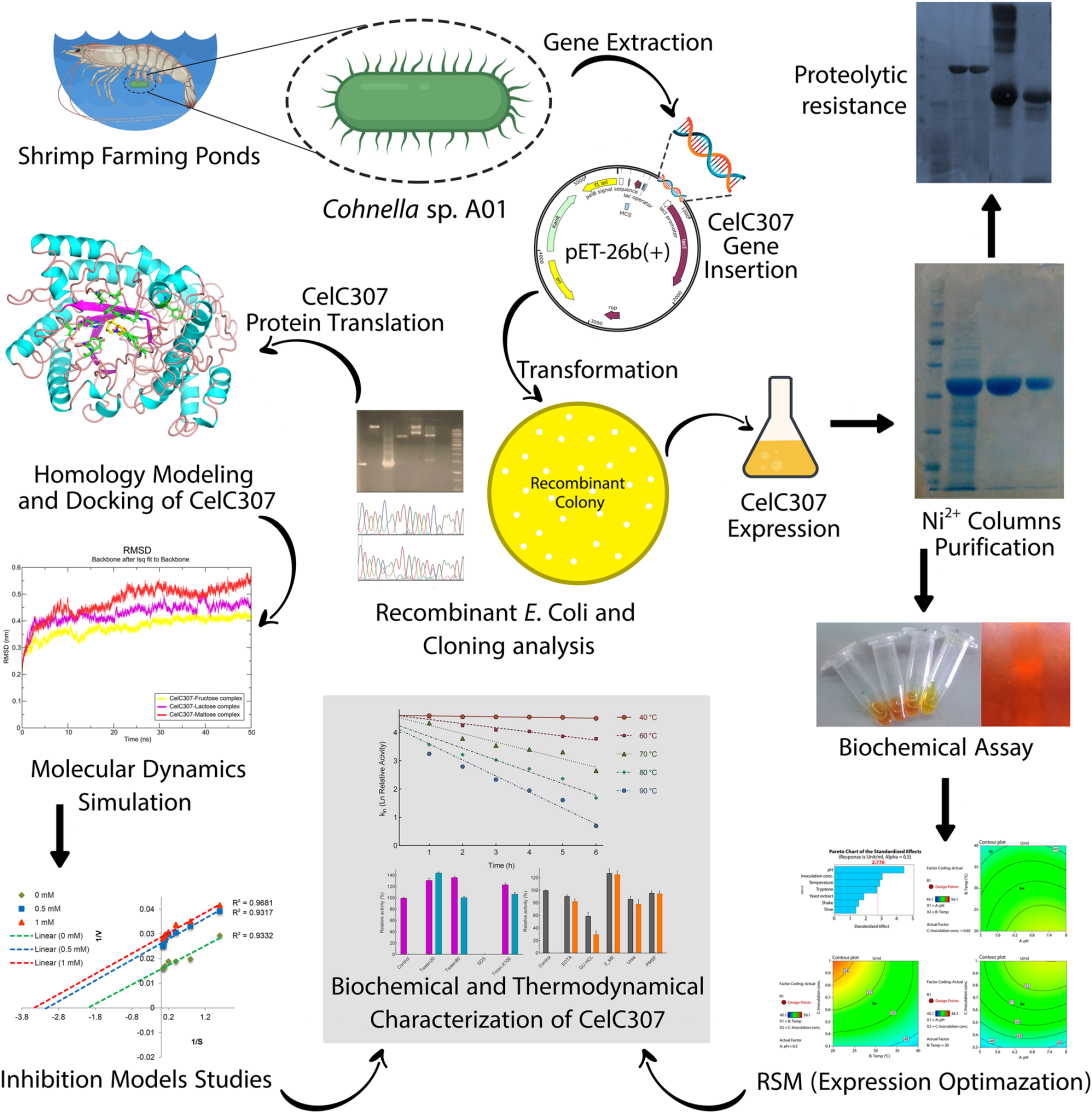
Flowchart summarizing the steps of CelC307 biochemical characterization and product optimization. Flowchart has been created by Biorender (BioRender.com) and Illustrator CS6.

The whole workflow that represents the overall procedures of biochemical characterization and product optimization of CelC307 is briefed in Fig. 1.

**Figure 2:**
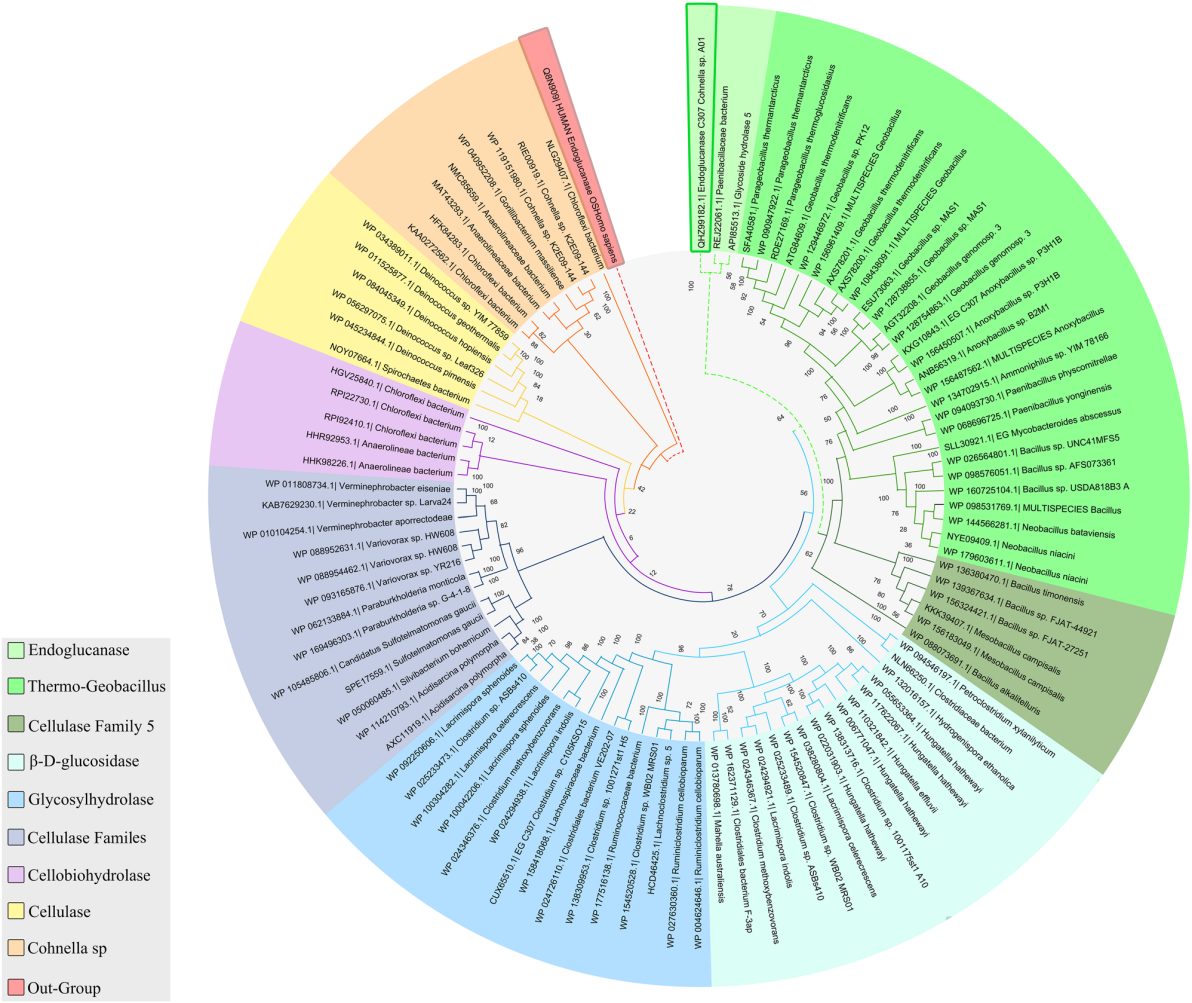
Phylogenetic analysis of CelC307. Unrooted circular phylogenetic tree of CelC307 from *Cohnella* sp. A01. The tree was constructed using MEGA 8.0 with the neighbor-joining method and it was modified and painted by Adobe Illustrator CS6. The CelC307 is marked with a green rectangle. Human endoglucanase (UniProt accession No. Q8N909) was used as the outgroup and is marked with a red rectangle. The color-coded branches correspond to the significant clusters.

The retrieved nucleotide sequence was translated into a protein with 491 amino acids. Computational analysis of the obtained protein sequence’s physicochemical qualities gave an overall idea about its nature and behavior. CelC307 theoretically was characterized as an intracellular endonuclease protein with 56.86 kDa molecular weight and pI of 5.85 that indicates the acidic nature of the molecule. The predicted stability index (37.17) for CelC307 was below 40, suggesting a stable protein. The computed aliphatic index and Grand average of hydropathicity index (GRAVY) were 69.80 and − 0.520, implying that the CelC307 is thermostable and hydrophilic, respectively. Phylogenetic tree analysis revealed that CelC307 has the maximum similarity to glycoside hydrolase from *Paenibacillaceae bacterium* (GenBank accession No. REJ22061.1) (Fig. 2), that according to the CAZy database, belongs to the large glycoside hydrolase family 5 (GH5) groups.

**Figure 3:**
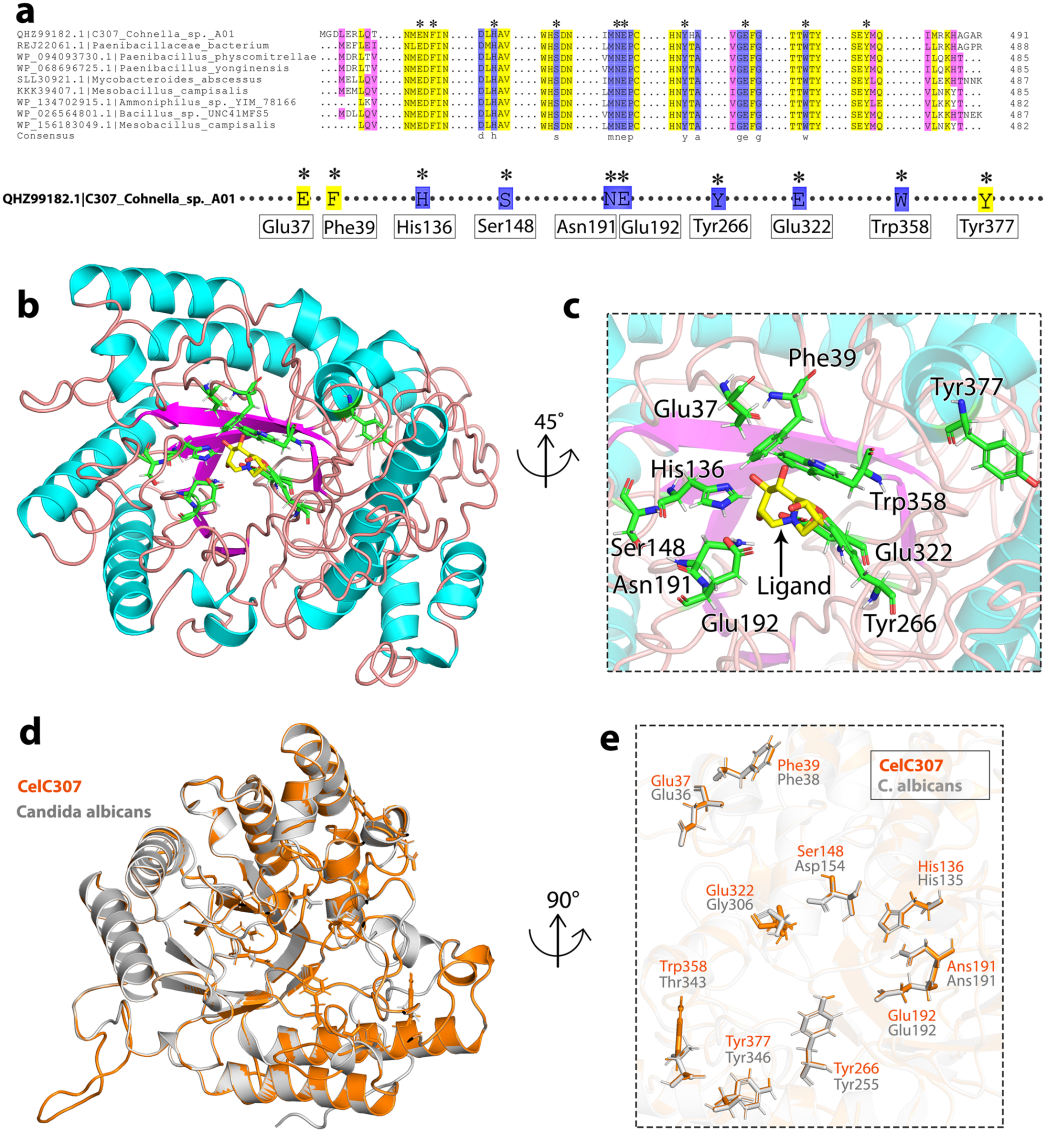
In silico structural characterization, and homology modeling of CelC307. (**a**) Multiple sequence alignments of CelC307 with cellulases from other genera and with the maximum of similarity. The blue shading indicates conserved identical residues. Yellow and pink shading indicates substitution with strongly and weakly similar residues, respectively. The predicted residues of the active site are star-marked. Sequence alignments were performed and visualized with DNAMAN software. (**b**) PyMOL V.2.3.4. pictured the CelC307 predicted protein model. The purple and blue structures are beta-sheet and alpha-helix, respectively. (**c**) PyMOL V.2.3.4. pictured the predicted active site structure in CelC307 protein. The amino acids colored in green are the residues in the active site interacting directly with the ligands (shown in yellow) selected by COFACTOR. (**d**) The 3D superimposition between the native structure of cellulase from *Candida albicans* (gray) and the predicted CelC307 structure (copper). The active site amino acids are featured in the image. (**e**) Adaptation of the active site amino acids of CelC307 and *Candida albicans* after superimposition.

CelC307 was anticipated to have a functional domain that included amino acids 77 to 362 based on prediction from the Conserved Domains server. The results of multiple alignments with some of the well-known cellulases (with the highest similarity) led to identifying the most conserved residues (Fig. 3a) (Full-length multiple alignments are included in Supplementary Fig. S1). The I-TASSER server modeled the CelC307 (I-TASSER idendity of 0.17) based on the template 3D structure of the exo-beta-(1,3)-glucanase from *Candida albicans* (PDB accession No. 1EQC). This template belongs to the GH5 superfamily and has a role in cell wall glucan metabolism and morphogenesis^8^. The predicted model of CelC307 was visualized using the PyMOL V.2.3.4. The modeled structure showed that CelC307 has the β-sheets at the center of the structure and α-helixes around the protein. Also, nuclear elbow motifs, containing protected glutamine and histidine, surround the active site of CelC307 and form a hollow cleft at the enzyme’s center (Fig. 3b).

The COFACTOR server predicted the active site residues (star-marked) (Fig. 3c) and compared them to the sequence alignment results. It is founded out that the predicted residues are conserved identical, except Glu37, Phe39, and Tyr377 that are among strongly similar residues. Structural superimposition of the template and constructed model revealed that the topology of the active site clefts and residues (predicted by COFACTOR, C-score: 0.19) in CelC307 (including Glu37, Phe39, His136, Ser148, Asn191, Glu192, Tyr266, Glu32^2^, Trp358, and Tyr377) has a strong match with the template (*Candida albicans*) (Fig. 3d,e).

### Docking results

**Figure 4:**
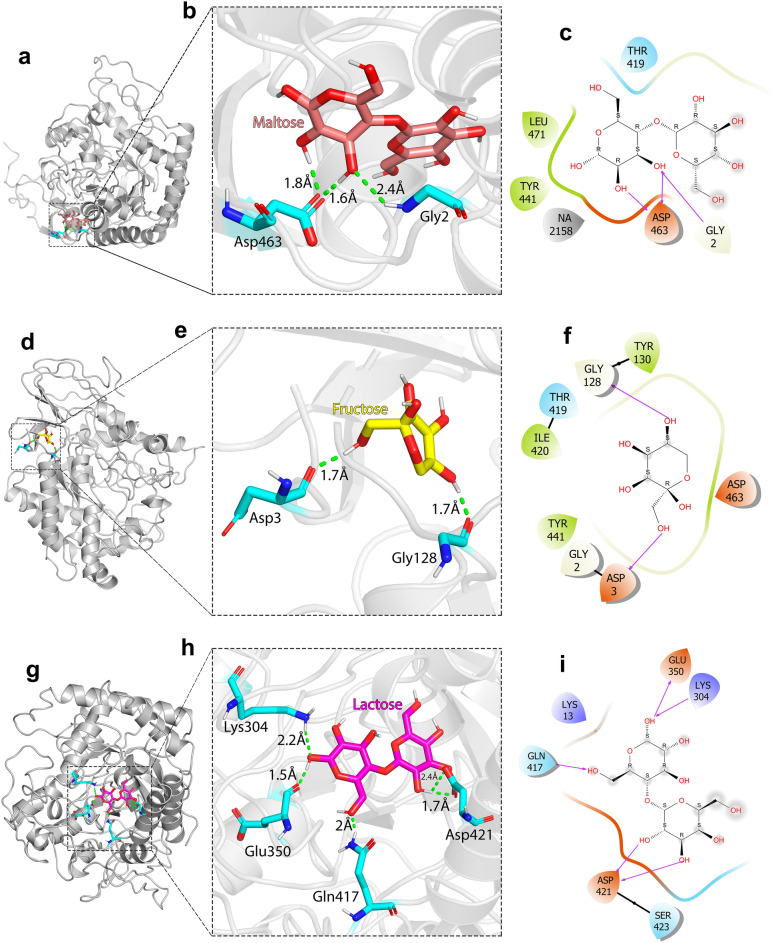
Docking results for CelC307 in complex with the sugar ligands. The complexes were pictured using PyMOL V.2.3.4. (**a**), (**b**) and (**c**) Docked CelC307-maltose complex and 2-D display interactions. (**d**), (**e**) and (**f**) Docked CelC307-fructose complex and 2-D display interactions. (**g**), (**h**) and (**i**) Docked CelC307-lactose complex and 2-D display interactions, after MD simulation.

Here we found the inhibitory effect of low concentrations of monosaccharides on the CelC307, and three of them (maltose, fructose, and lactose) were used for the docking study. The molecular docking analysis predicted amino acids involved in CelC307-ligands interaction (Fig. 4a,b,d,e,g,h).

Analysis of the docking results of the binding energy (after 50 ns of MD simulation) showed that the largest contribution of negative binding energy is reserved for amino acids that participate in hydrogen bonding with ligands. In contrast, the contribution of active site amino acids is very low. The results indicated that all three maltose, fructose, and lactose ligands contact with at least one Asp residue of CelC307 (Asp463 in CelC307-maltose complex, Asp3 in CelC307-fructose complex, and Asp412 in CelC307-lactose complex) by hydrogen bond in aqueous solutions (Fig. 4c,f,i). This may indicate the importance of Asp463, Asp3 and Asp412 in CelC307-sugars interactions.

### Molecular dynamics and MM/PBSA analysis

**Figure 5:**
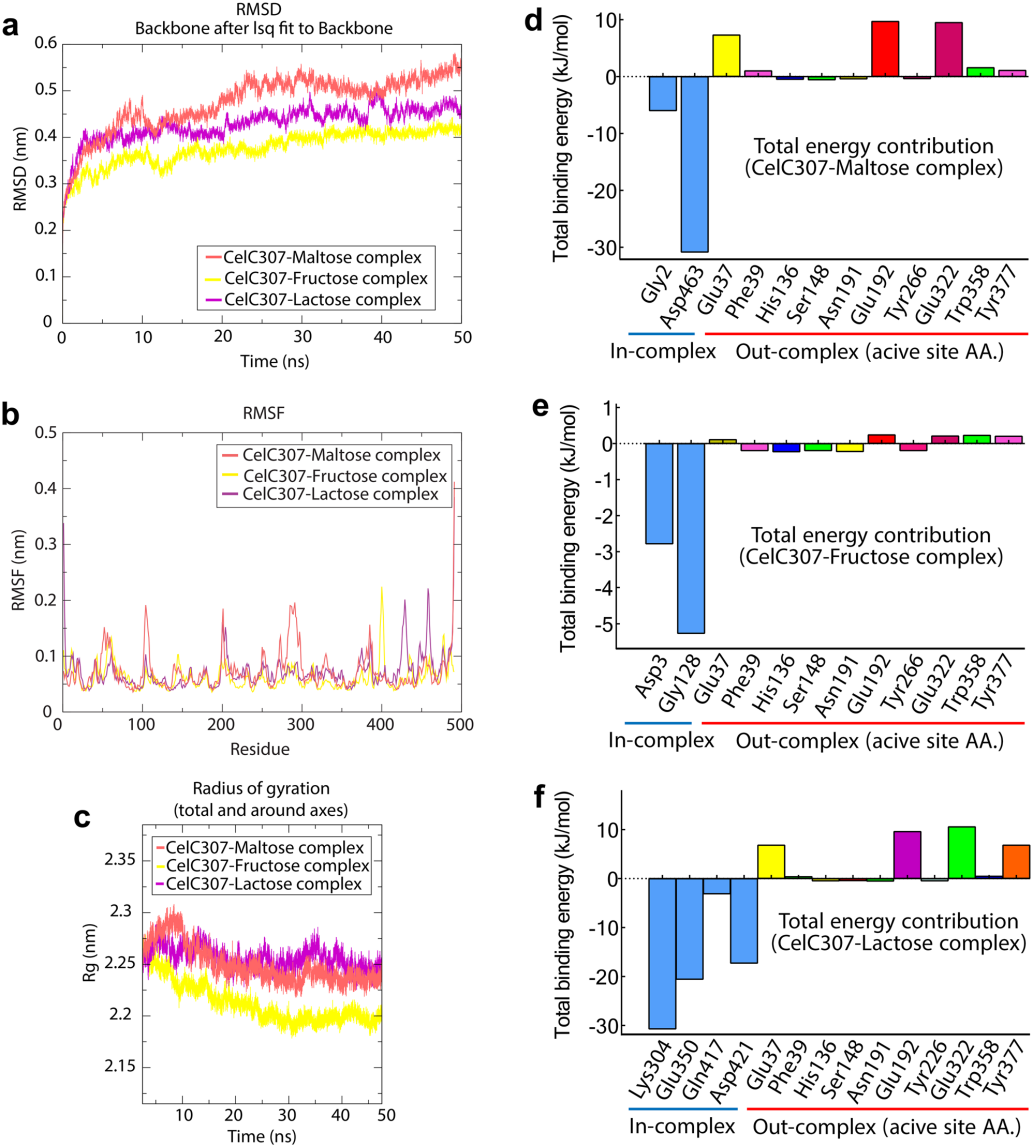
MD simulation, and MM/PBSA output of binding free energy contribution for CelC307 in complex with the sugar ligands. Grace software were used to draw (**a**) RMSD graph, (**b**) RMSF graph, and (**c**) Rg graph. The complex of CelC307 with maltose, fructose, and lactose are indicated with red, purple, and yellow colors, respectively. (**d**) The binding energy contribution of amino acids in CelC307-maltose, (**e**) CelC307-fructose, and (**f**) CelC307-lactose complexes, were drawn by GraphPad Prism V.8.

The docking results were simulated by MD to check the enzyme inhibitors’ stability and have atomically detailed structures. The low computed root mean square deviation (RMSD) values of docked CelC307-maltose, CelC307-lactose, and CelC307-fructose (0.42, 0.51, and 0.35 nm, respectively) (Fig. 5a) imply that the constructed models are robust and reliable for further analysis. To calculate the residual and side-chain flexibility, root means square fluctuation (RMSF) and radius of gyration (Rg) were calculated over 50 ns. The results demonstrated that all three sugars could destabilize the CelC307 structure; however, the less structural fluctuation was observed for CelC307-fructose when compared with the two other complexes (Fig. 5b). The Rg was calculated at 2.25 nm for CelC307 in complex with maltose and lactose. In comparison, the value was decreased in the CelC307-fructose complex (2.2 nm), which refers to the lower potential of fructose in CelC307 destabilization (Fig. 5c). However, it is assumed that the difference in Rg is not significant between complexes.

The binding free energy (kJ/mol) and corresponding components computed from the MM/PBSA analysis of CelC307-sugars complexes were calculated (Table 1). The obtained lower ΔG_binding_ supported the MD results for the CelC307-fructose complex, indicating the more favored energetically binding between fructose and CelC307. The MM/PBSA analysis results indicated that the electrostatic and then van der Waals interactions are the main contributor to binding in all three complexes. These calculated binding free energies support the corresponding results of MD simulation.

**Table 1:** Binding free energies and their corresponding components, obtained from MM/PBSA analysis of the CelC307 complexes with maltose, lactose, and fructose. All values are in kJ/mol.

Energy contribution	CelC307-maltose	CelC307-fructose	CelC307-lactose
ΔE_vdW_*	− 120.157 ± 12.917	− 74.466 ± 14.998	− 30.859 ± 40.318
ΔE_elec_**	− 287.955 ± 48.127	− 105.394 ± 14.821	− 299.667 ± 41.473
ΔG_polar_***	122.738 ± 40.386	106.216 ± 12.46	50.193 ± 19.4
ΔE_SAS_****	− 14.814 ± 0.867	− 10.007 ± 0.626	− 12.935 ± 0.728
ΔG_binding_	− 300.191 ± 20.192	− 83.651 ± 14.301	− 293.268 ± 23.756

*ΔE_*vdW*_ van der Waals interaction energy, **ΔE_*elec*_ electrostatic interaction energy, ***ΔG_*polar*_ polar solvation energy.

Each amino acid’s contribution to the binding energy is shown in Fig. 5d,e,f. It was found that the highest negative energy belonged to amino acids involved in complex interactions as well as the lowest negative energy belonged to CelC307 active site amino acids. These results support the idea of non-competitive inhibition of selected sugars.

### Heterologous expression, purification, and protease resistance of CelC307

**Figure 6:**
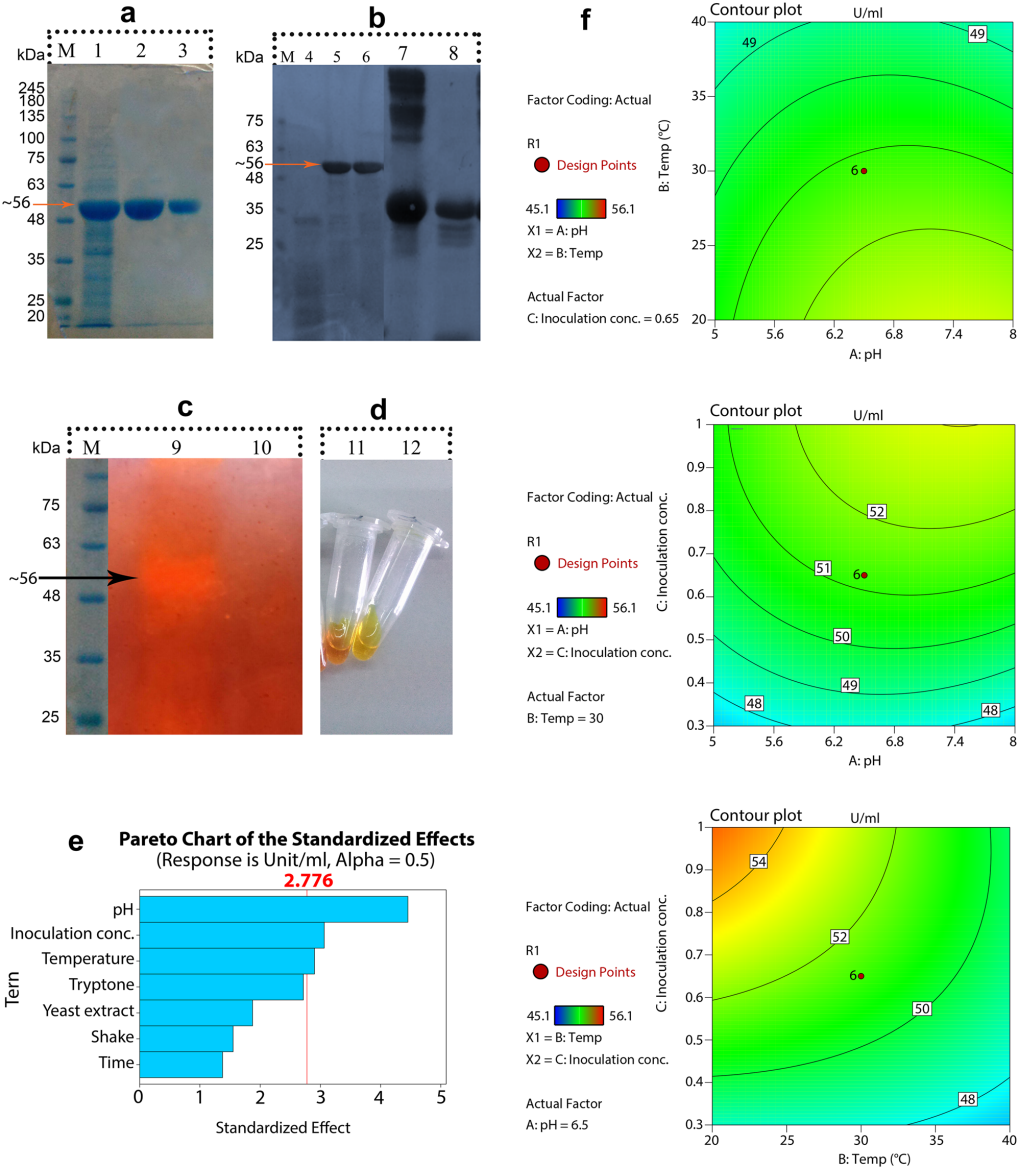
(**a**) M: protein marker, 1: crude extract of recombinant BL21, 2: Ni–NTA purified, 3: heat shock single-step method purified recombinant CelC307. (**b**) M: protein marker, 4: effect of proteinase K on CelC307, 5: effect of trypsin on CelC307, 6: purified recombinant CelC307, 7: BSA (positive control), 8: effect of trypsin on BSA. (**c**) M: protein marker, 9: formation of yellowish-orange halo related to the CelC307, and 10: empty pET26b(+) expressed in BL21 on the non-denatured gel. (**d**) 11: CelC307 activity assay (CMC 1% + enzyme), 12: blank control for CelC307 activity assay (CMC 1% + without enzyme). (**e**) Pareto chart of standardized effects shows the main effects of the variables, and response is cellulase activity (U/mg, α = 0.05) (obtained by Design-Expert 12). (**f**) The contour plots show the effects of variables and their interactions on cellulose activity (obtained by Design-Expert 12).

The amplified gene encoding CelC307 was cloned in the pET26b(+) vector under the control of the T7 promoter and with a His-tag. The cloned gene was confirmed by restriction enzyme digestion, colony PCR, and sequencing with gene-specific primers. The results of PCR and double digestion are provided in Supplementary Fig. S2. The SDS-PAGE analysis of the *E. coli* intracellular crude extract revealed the accumulation of recombinant CelC307 in the soluble form and with the approximate molecular weight of 56 kDa. The CelC307 enzyme purification was conducted using chromatographic and non-chromatographic methods; His-tag affinity to Ni–NTA and the single-step thermal shock. The homogenous purified enzyme, seen as a single protein band on SDS-PAGE, was obtained using both methods (Fig. 6a and Supplementary Fig. S3). Data on protein purification, including specific activity and purification fold, are summarized in Table 2.

**Table 2:** Data of CelC307 purification using Ni–NTA chromatography and single-step thermal shock.

Steps	Volume (ml)	Total protein (mg)	Total activity (U)	Specific activity (U/mg)	Purification (fold)	Yield (%)
Crude extract	4	6.4	82	12.8	1	100
Thermal shock	4	1.4	55	34.3	2.6	67
Ni–NTA	4	2.4	32.7	13.6	1.06	39

The purified CelC307 enzyme was mixed with proteinase K and trypsin and analyzed on the SDS-PAGE. 0.2% BSA was used as a control. While trypsin had a minimal effect on the CelC307, the digestion product of proteinase k was smeared on the SDS-PAGE. Both proteinase k and trypsin completely digested albumin (Fig. 6b and Supplementary Fig. S4).

### Substrate specificity and kinetic analysis

Cellulase activity was both quantitatively and qualitatively assessed. The Zymography test confirmed cellulase activity in the native-PAGE by creating an orange halo in the presence of CMC 1% substrate and Na^+^ 1 M (Fig. 6c and Supplementary Fig. S5). The value of cellulase activity was determined using CMC 1% substrate and detecting DNS color intensity (Fig. 6d and Supplementary Fig. S6). The highest levels of activity were observed for CMC substrate (*β-1 → *4 linkage) (100 ± 3.3%), followed by laminarin (21.6 ± 2.4%), chitin (8.4 ± 1.3%), pectic acid (1.9 ± 0.6%), and pustulan (NA) substrates. The kinetic parameters of CelC307 were computed through the Michaelis–Menten equation and using the CMC as the substrate. The values of K_m_, k_cat_, and k_cat_/K_m_ were determined 0.46 mM, 104.30 × 10^–3^ S^−1^, and 226.73 M^−1^ S^−1^, respectively. The kinetic parameters of CelC307 are compared with some of the other studied members of glycoside hydrolase family in Table 3.

**Table 3:** Comparison of activity, temperature, pH and kinetic parameters of CelC307 with other studied members of glycoside hydrolase family.

Source	Type/family	Substrate	Temp	pH	K_m_ (mM)	V_max_ (U/mg)	k_cat_ (S^−1^)	k_cat_/K_m_ (M^−1^ S^−1^)	Ref.
CelC307	Endoglucanase	CMC	40 °C	7	0.46	62.58	104.30	226.74	This study
CH43	EG family 5	CMC	65 °C	5	1.5	0.93	–	–	^9^
HR68	EG family 5	CMC	70 °C	6.5	1.7	1.70	–	–	^9^
*Bacillus subtilis* CBS31	Endoglucanase	CMC	50 °C	7.5	0.0183	1293.33	484,998	–	^10^
*Caldibacillus cellulovorans*	Cellulase	CMC	80 °C	6.5	3.4	44.7	–	–	^11^
*Bacillus vallismortis *RG-07	Cellulase	CMC	65 °C	7	1.923	769.230	–	–	^12^
*Bacillus subtilis* BC1	Cellulase	CMC	60 °C	8	1.243	271.3	–	–	^13^
*Penicillium simplicissimum *H-11	β-Glucosidase	CMC	60 °C	5	14.881	0.364	–	–	^14^
*Actinomyces* sp. KNG	Endo-β-1,4-glucanase	CMC	55 °C	7	0.39%	143	–	–	^15^
*Colletotrichum orchidophilum*	Endo-β-1,4-glucanase	CMC	55 °C	5	2.65	290.70	–	75.67	^16^
*Botrytis ricini* URM 5627	Endo-β-1,4-glucanase	CMC	50 °C	5	0.1299	0.097	–	–	^17^
*Aspergillus fumigatus* GH7	Endo-1,4-β-glucanase	CMC	55 ◦C	6	24.5	6193	5037	205.9	^18^
*Chaetomium thermophilum*	Endoglucanase7	CMC	55 °C	5	79.2	59.6	2.11	0.0267	^19^
*Myceliophthora thermophile*					24.0	622.5	–	0.313667	^20^
*Talaromyces emersonii* CBS394.64	Endo-1,3-,4-β-glucanase 7	CMC	70 °C	4.5	20.8	2257	–	78.9	^21^
*Aspergillus fumigatu*	Endo-1,3-1,4-β-glucanase 7	CMC	40 °C	4.5	209.7	51.9	43.3	0.2	^22^
*Daldinia eschscholzii*	Endoglucanase	CMC	70 °C	6	1.74	0.63	–	0.28	^23^
*Fusarium oxysporum*	Endo-1,4-β-d-glucanase	CMC	50 °C	6	23.2	22.5	–	0.55	^24^
*Melanocarpus* sp.MTCC 3922	Endoglucanases (EG I and EG II	CMC	50 °C	6	20.0	954	–	31.8	^25^
*Fomitopsis pinicola*	Endo-beta-1,4-glucanase family 5	CMC	50 °C	5	12.0	1250	–	55.4	^26^
*Penicillium purpurogenum*	Endo-1,4-glucanase	CMC	70 °C	5	1.15	220	–	118	^27^
*Gloeophyllum trabeum *CBS 900.73	Glycosyl hydrolase (GH) family 5	CMC	70 °C	4	4.5	1475	–	878	^28^
*Bacillus tequelensis* BD69	β-glucosidase	CMC	50 °C	5	7.43	1462	454.05	61.12	^29^
*Chaetomium thermophilum* CTendo45	Endoglucanase	CMC	60 ◦C	4	5.93	4.42	0.37	62.06	^30^

Optimization of culture condition.

In the first step of optimization, Plackett–Burman statistical method was used to find the most significant variables to obtain high cellulase activity (Supplementary Table S1). The obtained data showed that pH, inoculum solution concentration, and temperature positively affected the enzyme’s cellulolytic activity. The Pareto chart representing the main effects of variables is shown in Fig. 6e. These three variables were further subjected to the Box-Behnken design to optimize their magnitude, and other variables were excluded from the optimization. Using the Box–Behnken method, 20 sets of experiments with appropriate combinations of pH, temperature, and inoculation cell mass were conducted (Table 4). The cellulolytic enzyme activity (U/ml) was the dependent response variable. The highest cellulase activity in the Box-Behnken design was known to be 56.1 U/ml at 20 °C, and pH 7.5, and inoculation cell with an OD_600_ = 1. The mean observed and predicted responses for cellulose activity revealed that these data are in rational agreement and did not significantly differ.

**Table 4:** *Box–Behnken* experimental design and response values for cellulolytic enzyme activity (observed and predicted values).

Run	pH	Inoculum conc. (OD_600_)	Temp (°C)	Response )U/ml)	
				Actual	Predicted
1	5	0.3	20	47	46.91
2	8	1	40	49.3	49.10
3	6.5	0.65	30	49	51.01
4	5	0.3	40	46.2	46.51
5	6.5	0.65	30	51.4	51.01
6	5	1	40	47.6	47.84
7	8	0.3	20	48.5	47.96
8	8	1	20	56.1	55.50
9	6.5	0.65	30	51.9	51.01
10	5	1	20	52.1	52.44
11	8	0.3	40	46.4	45.76
12	6.5	0.65	30	50.6	51.01
13	6.5	0.65	13.18	52.86	53.23
14	9.02	0.65	30	49	50.02
15	3.97	0.65	30	48.7	48.07
16	6.5	0.65	30	51.7	51.44
17	6.5	0.65	30	52.4	51.44
18	6.5	0.06	30	45.1	45.52
19	6.5	0.65	46.81	47.5	47.52
20	6.5	1.23	30	53	52.98

A second-order polynomial equation was fitted to the response data obtained from the design, which resulted in the following regression equation [Eq. (14)]:

$${\text{R }} = \, + \;{2}0.0{4144} + {5}.{56251 }*{\text{ A }} + \, 0.{44628 }*{\text{ B }} + {17}.{38737 }*{\text{ C }} - \, 0.0{3}0000 \, *{\text{ A }}*{\text{ B }} + 0.{95238}*{\text{ A }}*{\text{ C }} - 0.{3}0000 \, *{\text{ B }}*{\text{ C }} - \, 0.{37659}*{\text{ A}}^{{2}} - {3}.{771}0{\text{6E}} - 00{3 }*{\text{ B}}^{{2}} - { 6}.{33976 }*{\text{ C}}^{{2}}$$

where R is the predicted response (cellulase activity), A is pH, B is temperature, and C is bacterial inoculation concentration.

The statistical significance of Eq. (14) was checked by the F-test and the analysis of variance (ANOVA) for the fitted (Table 5). The F-value of the model (15.34) and the associated *p*-value (*p* < 0.001) meant the regression model was significant. The F-value (0.6572) for the lack of fit was insignificant (*p* = 0.432), which confirmed the validity of the model. According to the *p*-values, the linear coefficients (B and C), two of the quadratic term coefficients (A^2^, C^2^), and one of the interaction coefficient (BC) had significant differences (*p* < 0.05), while other term coefficients were not significant. Figure 6f indicates the interaction effects of each of the two variables on the measured cellulase activity. To verify the predicted model, a culture medium with optimized predicted concentrations of independent variables was prepared. The maximum measured cellulase activity was found to be 62.58 U/ml, which authenticated cellulase activity of 58.4 U/ml as predicted by the RSM, and was ~ 3.1 fold more than the cellulolytic activity obtained in the basal condition. The obtained ‘Prob > F’ value for the model was < 0.0001, showing the model was statistically significant with a confidence interval of 99.99%. The results of a validation experiment under the optimum conditions verified the model predictions.

**Table 5:** ANOVA of Box–Behnken design to study the significance of factors and their interaction effect.

Source	Sum Of squares	df	Mean square	F-value	*p*-value	
Block	0.6135	1	0.6135			
Model	141.71	9	15.75	15.34	0.0002	Significant
A*	4.58	1	4.58	4.46	0.0640	
B**	39.46	1	39.46	38.44	0.0002	
C***	67.16	1	67.16	65.42	< 0.0001	
AB	1.62	1	1.62	1.58	0.2407	
AC	2.00	1	2.00	1.95	0.1963	
BC	8.82	1	8.82	8.59	0.0167	
A^2^	10.34	1	10.34	10.07	0.0113	
B^2^	2.05	1	2.05	1.99	0.1915	
C^2^	8.69	1	8.69	8.46	0.0174	
Residual	9.24	9	1.03			
Lack of fit	4.17	5	0.8334	0.6572	0.6759	Not significant
Pure error	5.07	4	1.27			
Cor total	151.57	19				

*A: pH, **B: temperature, ***C: inoculation concentration.

### Temperature and pH profile of activity and stability of CelC307

**Figure 7:**
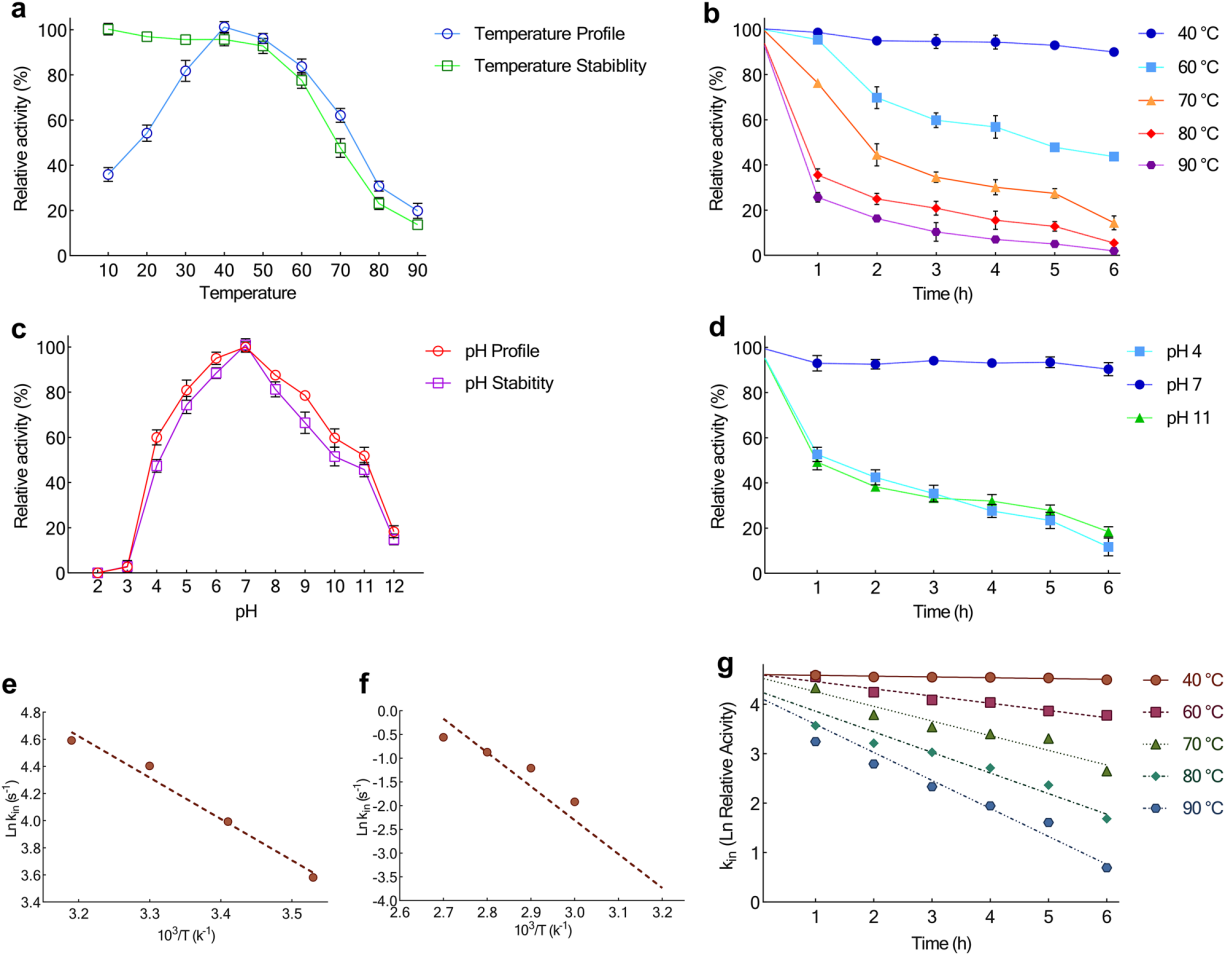
Effect of temperature and pH on the recombinant CelC307. Effect of temperature on the recombinant enzyme (**a**) activity and survival, and (**b**) stability. Effect of pH on the recombinant enzyme (**c**) activity and survival, and (**d**) stability. (**e**) Arrhenius plots for E_a_^‡^. (**f**) Arrhenius plots for E_a_^#^. (**g**) Thermal inactivation Ln at 60, 70, 80, 90, and 100 °C. The graphs were plotted by using GraphPad Prism V.8.

The maximum enzyme activity was detected at 40 °C with a specific activity of 13.6 U/mg. The assay of survival ability of CelC307 indicated that pre-incubation of the enzyme in temperatures higher than 50 °C, prior to its examination, led to a considerable decrease in its activity (Fig. 7a). Figure 7b exhibits the thermostability of the CelC307. The enzyme preserved more than 95% and 75% of its activity after thermal treatment at 60 and 70 °C, respectively. However, sharp declines were observed then after.

The activity and stability-pH profiles of CelC307 are shown in Fig. 7c and d. The enzyme exhibited optimal activity at pH 7. The pH survival profile of the enzyme was quietly compatible with the pH activity profile. In acidic and basic pH, the enzyme showed a considerable decay of activity.

### Thermodynamic analysis of the CelC307

The Arrhenius equation was used to calculate the activation energy of CelC307 (Fig. 7e). The values of thermodynamic parameters are shown in Table 5. The CelC307 needs 25.36 kJ/mole energy to activate the reaction (E_a_^‡^), which indicates that the reaction is rapid. Low ΔG^‡^ levels in CelC307 indicate the faster reaction for CelC307. On the other hand, the higher values of ΔH^‡^ and ΔS^‡^ at the optimum temperature emphasize the efficient transition state of CelC307. Also, compared to ΔG^‡^_E−S_ (2.01), lower values of ΔG^‡^_E−T_ (− 14.11) verified that a small value of energy can form the CelC307 TS at 40 °C, which suggests the possibility of the spontaneity of the reaction.

The CelC307 had very low k_in_ and, on the other hand, high t_1/2_ and D value at the optimum temperature (Fig. 7f,g). The highest t_1/2_ was obtained at 40 °C, but a considerable decrease and an increase were observed in the t_1/2_ and D values of higher temperatures, respectively.

The high E_a_^#^ value (59.29 kJ/mol) calculated for CelC307 confirmed that a high energy quantity is required for its irreversible thermal inactivation that is due to CelC307 inherent thermal stability.

The amount of ΔG^#^ for CelC307 increased with the temperature, indicating the enzyme’s resistance to irreversible thermal inactivation and denaturation, as well as the transition state that occurs later.

Enthalpy (ΔH^#^) and entropy (ΔS^#^) changes for deactivation of indicated decreasing trends with increasing temperature from 40 °C to 90 °C. The lower values of ΔH^#^ and ΔS^#^ at optimum temperature revealed a higher thermal stability of CelC307 at this temperature compared with the higher ones. ΔS^#^, ΔH^#^, and ΔG^#^ values revealed the thermophilic character of the CelC307 and indicated that this enzyme in the optimum temperature has a stable conformation. The thermodynamic characteristics of CelC307 are listed in Table 6.

**Table 6:** Thermodynamic parameters for the activation energy of CelC307.

Parameters	Value
E_a_^‡^ (kJ mol^−1^)	25.36
ΔG^‡^ (kJ mol^−1^)	58.60
ΔH^‡^ (kJ mole^−1^)	22.75
ΔS^‡^ (J mol k^−1^)	114.51
ΔG^‡^_E–T_ (kJ mol^−1^)	− 14.11
ΔG^‡^_E–S_ (kJ mol^−1^)	2.01
K_a_ (1/K_m_)	2.17

The parameters were measured after 6 h incubation at various temperatures, and the values were obtained at 40 °C.

The thermodynamic characteristics of CelC307 are compared with other studied endoglucanases in Table 7.

**Table 7:** Thermodynamic parameters for irreversible thermal inactivation of CelC307.

Temp.	k_in_ (m^−1^)	t_1/2_ (min)	D value (min/m^−1^)	E_a_^#^ (kJ/mol)	ΔH^#^ (kJ/mol)	ΔG^#^ (kJ/mol)	ΔS^#^ (J mol K^−1^)	Ref.
40 °C	1.62 × 10^–2^	427.86	1606.34	59.29	56.69	87.58	98.72	This study
60 °C	14.60 × 10^–2^	47.47	189.62		56.52	87.08	91.76	
70 °C	29.70 × 10^–2^	25.76	96.01		56.44	87.67	91.05	
80 °C	41.68 × 10^–2^	16.63	70.41		56.43	89.24	93.05	
90 °C	56.67 × 10^–2^	12.23	53.25		56.27	90.83	95.20	
								Other cellulases
44 °C	0.4 × 10^–2^	1732	–	378	375.36	111.36	833.06	^31^
50 °C	1.03 × 10^–2^	67.28	223.59	76.74	74.06	12.63	190.10	^32^
50 °C	1.49 × 10^–2^	46.51	154.56	62.80	60.12	53.10	21.72	^32^
60 °C	0.1 × 10^–2^	6930	–	256.81	254.04	118.69	406.45	^33^
25 °C	1.1 × 10^–2^	63.0 (h)	209.4 (h)	17.7	15.2	104.4	− 0.30	^34^
25 °C	1.7 × 10^–2^	40.8 (h)	135.5 (h)	66.3	63.8	103.4	− 0.13	^34^
50 °C	1.6 × 10^–2^	433	–	100.93	98.24	96.04	6.81	^35^

The parameters were measured after 6 h incubation at various temperatures, and the values obtained at 40 °C compared with other studied endoglucanases.

### Effects of metal ions, denaturing, inhibitors, detergents, organic solvent, surfactants, and Na^+^ on the activity of CelC307

**Figure 8:**
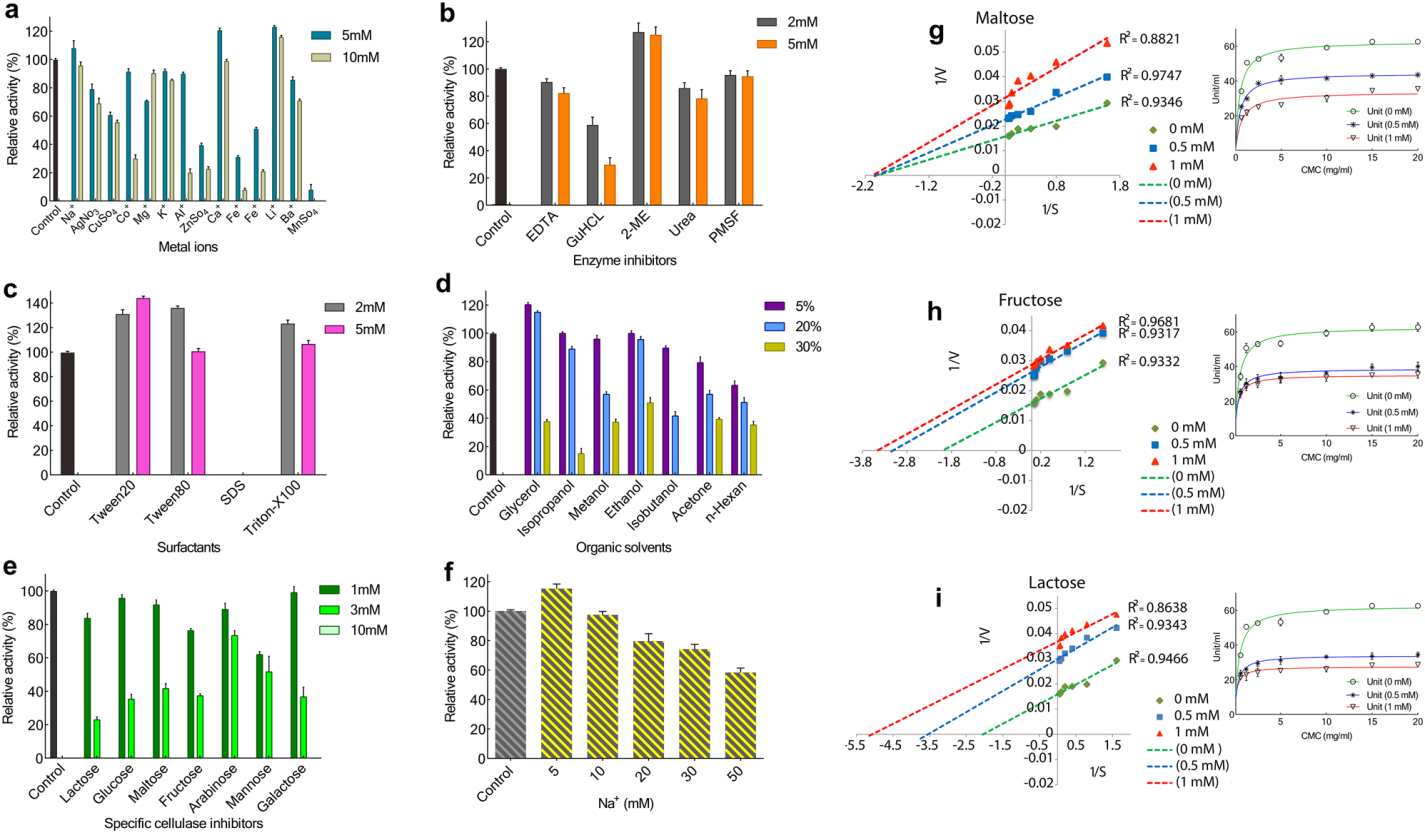
Effect of effectors on the recombinant CelC307. Effect of different (**a**) metal ions, (**b**) enzyme inhibitors, (**c**) surfactants, (**d**) organic solvents, (**e**) specific cellulase inhibitors, and (**f**) concentrations of Na^+^ on the CelC307 activity. The graphs were plotted by using GraphPad Prism V.8. Lineweaver–Burk plot of (**g**) maltose, (**h**) fructose, and (**i**) lactose. Lineweaver–Burk plots were drawn in Excel 2016. Each point represents the mean of three experiments. The cellulolytic activity was measured in the absence and presence of inhibitors.

We examined a list of potential activators or inhibitors of cellulases for their effects on CelC307 (Fig. 8a–f). Here, we found the significant inhibitory effect of most tested metal ions, especially ZnSo_4_, Fe^3+^, Fe^2+^, and MnSO_4_. However, Na^+^ and Ca^3+^ at a concentration of 5 mM, and Li^+^ at both tested concentrations (5 and 10 mM) slightly increased the activity of CelC307.

GuHCl as a denaturing agent displayed the highest inhibitory effect on the enzyme among the tested general enzyme inhibitors, while 2-ME showed the stimulatory effect. SDS was the only tested surfactant that completely inhibited the enzyme activity. In organic solvent, glycerol increased the CelC307 activity in concentrations of 5% and 20%. Increasing the concentrations of specific cellulase inhibitors up to 10 mM completely stopped the enzyme activity.

The CelC307 was found to well tolerate the low concentration of organic solvents (< 5%), and its activity was enhanced by up to 20% glycerol.

### Kinetic analysis of the specific inhibitors’ mode of inhibition and inhibition model

The inhibition mode of the CelC307 enzyme was measured with two concentrations (0.5 and 1 mM) of three substrates with the highest inhibitory effects; fructose, lactose, and maltose (Fig. 8g,h,i). Michaelis–Menten and Lineweaver–Burk plots were applied to determine the mode of CelC307 inhibition.

According to the obtained data, the inhibition mode of maltose was non-competitive because the V_max_ was reduced during the addition of inhibitor, while the x-axis and slope lines of the Lineweaver–Burk plot cross at the same point, and the K_m_ remained unchanged. This result indicated that the maltose could bind both to the free CelC307 enzyme and the enzyme–substrate complex. Furthermore, the fructose and lactose exhibited the mixed-type inhibition mode since, in the Lineweaver–Burk plots, the slopes cross at different points. Furthermore, the K_m_ and V_max_ values of the control and two concentrations of inhibitors were different. These results demonstrated that fructose and lactose could bind the enzyme if the substrate had already been bound.

## Discussion

Enzymes can generate biofuels-the fuels of the future-faster and cheaper than conventional chemical methods. Besides other glycolytic enzymes, cellulases act on cellulosic substrates to produce a cocktail of carbohydrates that can be converted to ethanol for biofuel. The increasing industrial demands for cellulases led to a growing interest in the engineering of existing cellulases and screening new sources to produce enzymes with improved performance. A broad range of microorganisms, including fungi, bacteria, and actinomycetes, have been recorded to be efficient cellulase enzyme producers. So, nature has gifted us a large number of cellulase enzyme sources with different characteristics and for various applications^36–38^.

In the present study, we identified the gene of a new cellulase from thermophilic indigenous *Cohnella *sp. A0.1, named CelC307, and determined the enzyme characterization using both experimental and computational approaches. In silico analysis of the translated sequence characterized CelC307 as an intracellular protein with thermostable, hydrophilic, and acidic nature. Multiple sequence alignment and phylogenetic analysis showed the high similarity between CelC307 amino acid sequence with glycoside hydrolase from *Paenibacillaceae bacterium,* classified in GH5 superfamily. GH5 is the first described and one of the most crowded GH families, classified by Aspeborg et al., into 51 distinct subfamilies^39^. GH5 members are found widely distributed in archaea, bacteria, and eukaryotes, and many enzyme activities relevant to biomass conversion have been found in this superfamily. GH5 enzymes use the classical Koshland two-step double-displacement mechanism for the catalysis, with the two catalytic residues (glutamates and histidine) at the C-terminal ends of β-strands 4 and 7^40^.

Commonly, cellulases have modular structure, containing a cellulose-binding module (CBM) and a core, catalytic domain (CD) linked via a flexible, frequently glycosylated linker^41^. According to the Conserved Domains server, the Celc307 CD is predicted to contain amino acid regions 77–362, and CBM is likely to be found in the enzyme's C-terminal region. The hydrolyzing activity of CelC307 is attributed to several amino acids in the active site, including tyrosine that plays a role in binding to the sugar chain, and aspartic acid and glutamic acid that are catalytic amino acids responsible for glycosidic bond cleavage (nucleophilic attack). Furthermore, it has been reported that arginine, asparagine, and histidine are among the highly conserved amino acids at the active site of cellulases GH5^42,43^. These motifs probably provide the enzyme for easy access to the substrate^44,45^.

It was shown that high concentrations of individual monosaccharides (mannose, glucose, galactose, xylose, and fructose) have a non-competitive inhibition effect on the hydrolysis of commercial cellulase cocktails^46^. Docking results showed three ligand molecules bind at different places other than the active site that suggested the non-competitive inhibition mechanism. Furthermore, ligands contact with at least one Asp residue of CelC307(Asp463, Asp3 or Asp412) by hydrogen bond in every three complexes may indicate that Asp in CelC307-sugars interactions is a key amino acid.

The overall results of MD simulation showed the CelC307 protein instability in the presence of all three tested sugars after 20 ns; however, the lower calculated RMSD, RMSF, and Rg indexes for CelC307-fructose implied the lower instability for CelC307-fructose complex. The MM/PBSA calculations were carried out to determine the binding free energy between CelC307 with the three selected sugars. It was found that the electrostatic and van der Waals interactions have principle contribute to the formation of interactions in all three complexes. Hydrogen bonding is the main stabilizing force in protein stability and plays a key role in maintaining structural integrity. The amino acids involved in hydrogen bonding showed the largest negative free energy, while those that were predicted in the active site showed the lowest contribution in binding. These results support the idea of non-competitive inhibition of selected sugars.

The CelC307 enzyme purification was conducted using chromatographic and non-chromatographic methods; His-tag affinity to Ni–NTA and the single-step thermal shock. However, the single-step thermal shock is fast, easy, cost-effective, and interestingly led to a remarkable higher yield for this purified protein. In the previous study, we purified the protease 1147 from the same microorganism using the single-step thermal shock with ~ 73% efficiency^47^. So, it seems this simple introduced method could be considered a practical technique for isolating thermostable protein, especially for industrial enzymes.

We examined the effects of two proteases (trypsin and proteinase k) on CelC307. It was found that CelC307 is well resistant to trypsin, while proteinase k digested the CelC307 completely. Therefore, in the case of using this enzyme in animal feed, trypsin is suggested as the supplemented protease. Studies have shown that the use of exogenous enzymes in animal diets may be influenced by ruminal proteolytic enzymes. Since the use of cellulases in ruminants diets such as cattle is very common, stability against proteolytic digestion can be a significant benefit for exogenous cellulase enzymes^48,49^. An important application of cellulases enzymes is in the animal feed industry, which, alongside other enzymes, including proteases, improves feed utilization and animal performance^50,51^.

CelC307 showed the highest cellulolytic activity against soluble CMC substrate (β-1 → 4 linkage) used to calculate kinetic parameters. Compared to some other studied cellulases^19,52–55^, CelC307 showed low K_m_ and high k_cat_/K_m_, indicating a high preference of the enzyme for CMC that led the enzyme to hydrolyze *β-*1* → *4 linkages more rapidly than other evaluated cellulases.

Economic aspects are the main barrier to the enzymes’ application on the large-scale. To optimize the production processes, the other aim of this study was to evaluate the parameters involved in the production of recombinant CelC307 from *E. coli* using statistical designs. We used the Plackett–Burman, and Box–Behnken design to provide accurate results for increasing the production of recombinant CelC307. Compared to other RSM designs, Box-Behnken has three levels and requires fewer experiment runs, making it easier to arrange and interpret^56^. The successful application of RSM methodology for optimizing cellulase enzymes has been reported in different studies^57,58^. Jeya et al. used the statistical experimental design to optimize the hydrolysis parameters (such as temperature and pH) of cellulase from fungus *Trametes hirsuta* and achieve an ~ 18% increase in the saccharifying rice straw^59^.

Overall, the results suggest that CelC307 can tolerate a broad range of temperature and pH. Fermentation of lignocellulose materials in the bioethanol industry and alcoholic fermentation usually proceeds at low temperature and natural pH (37 °C and pH 7)^60^, which correspond to the features of CelC307.

Furthermore, neutral and alkaline cellulases find application in many other industries such as food, brewery, and wine. Most isolated cellulases with fungal sources have acidic pH optima, so screening for neutral and alkaline cellulases, usually from bacterial sources, is of great interest for biotechnological research^61^.

Thermodynamic relationships were used to demonstrate the enzyme’s ability to maintain activity at high temperatures and the inherent structural stability of CelC307. The E_a_ of the enzyme was obtained by calculating the thermodynamic parameters of conversion [S] and [E] to [ES]. The amount of energy needed to start a reaction is called E_a_. The lower the energy of the reacting molecules, the faster the reaction occurs. The spontaneity of the catalytic reaction is demonstrated by ΔG and the effectiveness of the transition state (TS) by ΔH and ΔS^62^. In general, the thermodynamic parameters of ES complex formation showed that CelC307 had lower values of E_a_^‡^, ΔG^‡^, and ΔG^‡^_E–T_ compared to ΔH^‡^, ΔS^‡^, and ΔG^‡^_E–S_. These results proposed that the CelC307 reaction is likely to be faster and has an efficient transition state. Similar results have been reported in two studies related to the endoglucanase cmc-1 and CBS31 obtained from *Aspergillus oryzae* and *Bacillus subtilis*^10,31^.

The basis for calculating the thermodynamic parameters of irreversible thermal inactivation is to obtain the k_in_, t_1/2_, and D value. A decrease in T_1/2_ and D value and an increase in k_in_ during the temperature rise is a consistent hypothesis about thermostable enzymes. This fact has also been demonstrated in several heat-resistant endoglucanases^33,35^.

Parameters of the irreversible thermodynamic can be determined by the transition state (T) calculation of protein. It is assumed that irreversible denaturation of proteins is a two-step reaction: N ↔ U → I, that the transition state refers to a protein formed between N (native state) and U (reversible/partially unfolded state), or I (irreversible/inactivated state)^63^. A key factor in understanding an enzyme’s thermal capacity is calculating the activation energy needed for an enzyme’s irreversible thermal inactivation.

CelC307 showed a high E_a_^#^ and ΔG^#^ value in its irreversible thermal inactivation process. In the thermodynamic study of three thermophilic endoglucanases from *Aspergillus oryzae*, *Macrotermes subhyalinus*, *Aspergillus fumigatus*, and a β-glucosidase enzyme from *Aspergillus niger*, a similar increase in ΔG^#^ was observed in the transition state with the increasing temperatures^31–33,64^. On the other hand, the enzyme’s stability and resistance against the denaturation process are associated with maximum ΔS^#^ and ΔH^#^ at the optimum operating temperature^65^. A decreasing trend of ΔH^#^ and ΔS^#^ at higher temperatures indicates the change in CelC307 structure toward the transition state^66^. However, high values of ΔG^#^ and E_a_^#^ indicate that the CelC307 requires a large amount of thermal inactivation energy for denaturation, so, CelC307 resists the transition state occurrence. Comparable results in transition state values (E_a_^#^ and ΔG^#^) have been observed for cellulase enzyme^34^. In general, these findings indicate the thermal resistance of CelC307, in the TS phase, at high temperatures. These results can provide an insight that shows the stability of CelC307 at the TS phase can be inherited from its catalytic efficiency^66^.

The enzyme activities may be affected by different reagents employed in different industrial processes or may be formed due to equipment erosion or corrosion^67^. So, identifying the knowledge about the CelC307 activators and inhibitors is quite relevant, essentially for further evaluation of its industrial application. Metal ions exert their effect by associating with enzymes or making a complex with other molecules linked to enzymes serving as electron donors or acceptors, Lewis acids, or structural regulators^68^. It seems that ions affect cellulases from various sources differently. However, similar results were reported for the endoglucanase isolated from *Aspergillus terreus*^69^. The observed decreased enzyme activity in the presence of most of the examined metal ions suggested that CelC307 is not a metalloenzyme, however, its activity is affected by metals. The results showed that Triton X-100, Tween 20 and Tween 80 increased CelC307 activity. Surfactants have a great affinity for interphases due to their dual nature and amphiphilic structure, which is the basis of the methods by which surfactants impact the physicochemical properties of proteins^70,71^. Tween 20 is supposed to act as a chemical chaperone, assisting in protein folding by direct hydrophobic interactions with proteins and altering protein interactions with surfaces^72^.

Glycerol has been known as the enzyme stabilizing agent that can shift the native protein ensemble to a more compact state. It also can inhibit protein aggregation by preventing protein unfolding^73^. This result is quite notable, considering the potential application of CelC307 for processes that perform in the presence of organic solvents^74^. For instance, the pretreatment of biomass with crude glycerol was reported to improve the enzymatic hydrolysis of cellulose^75^. The addition of 2 and 5 mM of EDTA had a slight inhibitory effect on the CelC307 activity. Similar observations were also made for CelRH5, and C67-1 derived from rhizosphere and buffalo rumen (both from the GH5 family)^76,77^.

Overall, CelC307 seems to be a stable enzyme with no substantial reduction in its activity upon adding some well-known enzyme inhibitors, including PMSF, urea, and 2ME, and non-ionic surfactants. A significant reduction in the CelC307 activity was only observed by the addition of GuHCl. Also, the anionic surfactant SDS completely inactivated CelC307 even at low concentrations (2 mM), which was reported previously for other cellulases GH5 family^76^.

Supplementation of media with different Na^+^ concentrations indicated that CelC305 remained 70% of its activity up to a 50 mM Na^+^ concentration, and a low concentration of Na^+^ (5 mM) has an enhancing effect on its activity. Also, Na^+^ resistance has been reported in an acidic cellulase from buffalo rumen metagenome (20 mM) and endoglucanase of extreme halophilic *Haloarcula *sp. CKT3 (3 M)^78,79^. Salt tolerance is a special feature of this novel endoglucanase that could indicate the potential of using CelC307 in the food industry.

Overall, the thermostable CelC307 can well tolerate the presence of various additives used in different industries, making this novel isolated cellulase a promising candidate for further research and industrial applications.

## Conclusion

This study reports a novel cellulase (CelC307) from the thermophilic indigenous *Cohnella *sp. A01 that efficiently catalyzes the hydrolysis of CMC substrate (β-1 → 4 linkage). The production of recombinant CelC307 was optimized using RSM methodology and reached 62.58 U/ml. The thermodynamic parameters calculation verified the inherent thermostability of CelC307. The molecular docking and MD analysis suggested that inhibitors bind at different places other than the active site and in the non-competitive inhibition mechanism. According to the characterization results, CelC307 could be considered a promising candidate for further research and industrial applications.

## Methods

### Strains, vector, and chemical reagents

High pure DNA and vector purification kit were supplied from Peqlab and Roche (Germany). High pure PCR product purification kit was purchased from Bioneer (Korea). Vector pET26b(+) and enzymes *Xoh*I, *Nde*I were gained from Fermentas (Germany). The *E. coli* DH5 α BL21 (DE3) strains were obtained from Invitrogen (USA). Carboxymethyl cellulose (CMC) and other chemicals were supplied from Merck (Germany).

### Software

GraphPad Prism (V.8 GraphPad software, USA, https://www.graphpad.com/) was applied to draw the Michaelis–Menten curve, calculate the K_m_ and V_max_, and the effect of temperature and pH. PyMOL V.2.3.4 (https://pymol.org/) was utilized for visualizing the protein structure. Protein–ligand docking was performed using AutoDock Vina V.4 in Chimera V1.13.1 (https://www.cgl.ucsf.edu/), and molecular dynamics simulations were conducted using GROMACS V4.6.5 (https://www.gromacs.org/).

### In silico structural, functional, phylogenetic analysis and homology modeling

The genome of *Cohnella sp*. Strain A01 was sequenced previously^80^, and the predicted sequence for endoglucanase cellulase C307 (CelC307) encoding gene was used in this study (GenBank accession No. MN105992.1). The Expasy service (http://web.expasy.org/translate/) was applied for translating the nucleotide sequence, and the ProtParam (https://web.expasy.org/protparam/) for the determination of physicochemical features of the predicted protein. The family classification of CelC307 was performed according to the CAZy database^81^. The highly conserved amino acid sequence of CelC307 with the other homologous enzymes was identified using DNAman software (https://www.lynnon.com/). The neighbor-joining phylogenetic tree was drawn by MEGA 8.0 (https://www.megasoftware.net/), and human endoglucanase (UniProt accession No. Q8N909) was used as the outgroup.

I-TASSER server was used to build CelC307 3D structure by providing amino acids sequence^82^. Endoglucanase of *Candida albicans* (PDB accession No. 1EQC) was known as the template crystallographic model as the best protein template for the construction of CellC307 3D. The generated model was refined in the ModRefiner service^83^. The information about the active site of the CelC307 enzyme was obtained from COFACTOR^84^. The alignment of PDBs was made by TM-align^85^. Functional domain of CelC307 was identified via Conserved Domains server (.https://www.ncbi.nlm.nih.gov/Structure/cdd/wrpsb.cgi).

### Docking of CelC307-inhibitors

The modeled CelC307 was docked against three cellulase-specific inhibitors (maltose, fructose, and lactose), which showed the highest inhibitory effect on CelC307 enzymatic activity. All of the surrounding water molecules were removed, and an energy minimization step was performed to obtain a stable CelC307 structure. The PubChem server (https://pubchem.ncbi.nlm.nih.gov/) was used to retrieve the chemical structures of fructose, lactose, and maltose, and AutoDock Vina was used for docking. The inhibitors were saved in the Mol2 format and were docked at the appropriate box with an adjusted complex grid size of 80 × 60 × 50 Å. Complexes were run and saved in the PDBQT formats, and each complex was analyzed by the ViewDock command in the Chimera V1.13.1.

All the docking results were shown by PyMOL V.2.3.4 and Maestro V11.8 in Schrödinger 2018.1 suite.

### Molecular dynamics simulation and MM/PBSA calculation

The prepared CelC307-ligand complexes were simulated using the molecular dynamics method using the open-source molecular dynamic package, GROMACS V4.6.5., with the CHARMM fully atomic force-field. The production run was 50 ns. The trajectories related to each complex were used for analyzing MD results by Grace software in the Bio-Linux 8 operation system.

The binding free energy of the simulated CelC307-ligand complexes was computed using the molecular mechanics/Poisson–Boltzmann surface area (MM/PBSA) method. 100 snapshot frames obtained from 50 ns of simulation, and the binding free energy (ΔG_binding_) was calculated according to the Eq. (1):

1 $$\Delta {\text{G}}_{{{\text{binding}}}} = \Delta {\text{G}}_{{{\text{binding}}}} \left( {{\text{vacuum}}} \right) \, + \Delta {\text{G}}_{{{\text{solvation}}}} \left( {{\text{complex}}} \right) \, {-} \, (\Delta {\text{G}}_{{{\text{solvation}}}} \left( {{\text{CelC3}}0{7}} \right) \, + \Delta {\text{G}}_{{{\text{solvation}}}} \left( {{\text{ligand}}} \right)$$

where ΔG_solvation_ is the sum of electrostatic (G_polar_) and non-electrostatic (G_apolar_) results of complexes.

### Cloning, expression, and purification of *Cohnella *sp. A01 CelC307

A colony of *Cohnella sp.* A01 was added to nutrient broth medium, cultured at 55 °C for 72 h, and centrifuged (8000×*g*, 20 min at 4 °C), then the harvested cells were used for DNA extraction. The predicted cellulase C307 gene was amplified by PCR, using the forward and reverse primers that incorporated *Nde*I and *Xoh*I restriction sites, respectively (For; 5′-GGAATTCCATATGGGAGATTTGGAACGGCTTC-3′, and Rev; 5′-CCGCTCGAGCCTTGCGCCCGCATG-3′). The reverse primer excluded the stop codon that led to incorporating the polyhistidine-tag located at the C-terminal of CelC307. The amplified fragment was cloned into the expression vector pET26b(+). The recombinant plasmid was transformed into *E. coli* BL21 (DE3) strain using the heat shock transformation method. The positive plasmids were identified by restriction enzymes digestion, PCR, and sequencing.

Kanamycin solution at the final concentration of 30 mg/ml was added to the LB medium. A single recombinant colony was dissolved in LB medium, after 16 h grown at 37 °C transferred into a fresh LB medium containing 1 mM IPTG, and were incubated at 37 °C to reach OD600 = 0.6. The cells were pelleted by centrifugation, resuspended in 4 ml of lysis buffer (50 mM), and subjected to sonication (4 cycles of 45 s pulse with one min interval on ice). The obtained supernatant from centrifugation of crude cell extracts (9000×*g*, 45 min at 4 °C) was used for protein purification. The purification step was performed via two different methods; (I) purification with Ni–NTA sepharose column chromatography was accomplished according to the procedures previously described^47^. (II) Purification with a heat shock method; the acquired supernatants were incubated at 70 °C for 10 min, followed by 10 min centrifugation at 10,000×*g*. The purified proteins were assayed for cellulase activity and subjected to SDS-PAGE (12%) for visualization. The Protein concentration was estimated using the Bradford’s method^86^.

### CelC307 activity and substrate specificity

According to the method described by Miller, cellulase activity is determined by the estimation of reducing sugars that release from the carboxymethylcellulose (CMC) substrate by using dinitrosalicylic acid (DNS) reagent^87^. Different concentrations of commercial cellulase enzyme and 1% (w/v) CMC substrate were dissolved in potassium phosphate buffer (pH 7) and were used to plot the standard curve. The enzyme-free reaction was used as a negative control. The enzymatic reactions were incubated at 40 °C for 5 min, then terminated by an equal quantity of DNS. 10 min incubation in the boiling water led to an alkaline mixture of reducing sugars with DNS, and a red color developed. The color intensity has a linear relationship with the concentration of reducing sugars. One unit of the cellulase enzyme is described as the enzyme’s quantity that liberates one μmol of reducing sugar per min.

The specificity of the CelC307 was determined by testing 1% (w/v) of Laminarin, Pustualn, chitin, and pectic acid as the substrate. We analyzed all reactions in triplicate.

### Zymography and protease digestion resistance

According to Schwarz et al., Zymography was performed using a non-denatured PAGE containing 0.1% CMC^88^. The gel was immersed at 4 °C for 30 min in 100 mM potassium phosphate buffer (pH 7), washed in distilled water, incubated for 15 min in Congo red staining solution, and finally washed with 1 mM Na^+^ solution. The clearance zones around bands corresponding to the purified CelC307 appeared gradually.

Resistance to proteolytic digestion was tested by two proteases; trypsin and proteinase k. One mg/ml of proteases and 0.6 mg/ml of CelC307 were mixed in a ratio of 20:1 (v/v) and incubated at 37 °C for 30 min. Digestion of bovine serum albumin (BSA) with the same ratio was used as the positive control. The results of digestion reactions were analyzed with SDS-PAGE.

### Michaelis–Menten kinetics

The affinity of CelC307 for CMC substrate was determined by calculating the enzyme activity in the presence of an increasing concentration of substrate (0.625 to 20 mg/ml), and the Michaelis–Menten equation was fitted to the data. The CelC307 kinetic parameters (K_m_, V_max_, k_cat_, and k_cat_/K_m_) were calculated by applying GraphPad Prism V.8 with nonlinear regression analysis using the Michaelis–Menten model.

### Optimization of expression

Statistical multivariate optimization (SMO) was used to obtain the optimal response (here CelC307 enzyme activity) by statistical analysis. The SMO was performed in two steps. Firstly, 12 sets of trials were conducted based on Plackett–Burman design in the screening experiment to determine the most critical factors affecting the CelC307 enzyme activity^89^. All factors were examined at two levels and consisted of; pH (3 and 8), temperature (20 and 40 °C), rotation (70 and 200 rpm), optical density (0.3 and 1), induced time (4 and 18 h), yeast extract (2.5 and 7.5 g/l), and Trypton (7.5 and 12.5 g/l). In the next step, the obtained key factors (here pH, temperature, and inoculation concentration) were further optimized using the Box-Behnken method to reach the best possible levels of the factors for the highest CelC307 enzyme activity^90^. Twenty experimental trials with six replicates at the center point were designed for the three selected factors. Each factor was investigated at five levels (between − 1 and + 1), and the response value was the CelC307 activity (unit/ml). Minitab 16 (Minitab Inc., USA, https://www.minitab.com/en-us/) and Design-Expert 12 (Design-Expert Inc., USA, https://www.statease.com/software/design-expert/) software were applied for experimental data analysis.

### Effect of temperature and pH on the CelC307 activity and stability

A temperature range of 10–100 °C, with 10 °C intervals, was applied to assess the effect of temperature on the CelC307 activity and stability. Thermal stability of the CelC307 was inspected in two ways: (I) the enzyme was pre-incubated at a temperature range of 10 to 100 °C (with 10 °C intervals) for 90 min and then assayed at optimum temperature, (II) the activity of CelC307 was assayed at 60 to 90 °C for 6 h and with one-hour intervals.

CMC 1% (w/w) substrate was dissolved in 50 mM acetate, phosphate, and glycine buffers that provided the acidic, neutral, and basic pHs in the range of 2 to 12. The enzymatic assay was performed at the optimum temperature (40 °C). Subsequently, the pH stability of the enzyme was assayed in two ways: (I) the enzyme was preincubated in a pH range of 2 to 12 for 90 min, then assayed in optimum condition, (II) the residual CelC307 activity was assayed at pHs of 4, 7 and 11 for 6 h and with the one-hour intervals. All assays were performed in triplicate.

### Effect of metal ions, detergents, inhibitors, organic solvents, Na^+^, and sugars on cellulolytic activity

The Effect of 5 and 10 mM concentration of Na^+^, AgNO_3_, CuSO_4_, Mg^2+^, K^+^, Al^3+^, ZnSO_4_, Ca^2+^, Fe^3+^, Fe^2+^, Li^+^, Ba^2+^, and MnSO_4_, as metal ion solutions, was investigated on the CelC307 activity. Meanwhile, the effect of potential enzyme inhibitors was determined in concentrations of 2 and 5 mM of chemical substances, including phenyl-methylsulfonyl fluoride (PMSF), β-mercaptoethanol (2-ME), guanidine hydrochloride (GuHCl), urea, and EDTA. The effect of surfactants on the CelC307 activity quantitation was determined using 2 and 5% (v/v) concentrations of SDS, Tween-20 and 80, and TritonX-100. The concentrations of organic solvents [5, 20, 30% (v/v)], including methanol, ethanol, isopropanol, isobutanol, glycerol, acetone, and n-Hexane, as well as Na^+^ at the final concentrations of 5, 10, 20, 30, and 50 mM, have been used in enzymatic reaction to finding out their catalytic effects. Furthermore, the effects of various sugars comprising galactose, mannose, arabinose, fructose, maltose, glucose, and lactose (as the specific cellulase inhibitors and with the concentrations of 1, 3, and 10 mM), have been investigated on the CelC307 enzyme activity. The CelC307 activity, without any additive in the assay reaction solution, was assumed control (100% activity).

### Thermodynamic study

Arrhenius equation was used for the calculation of CelC307 activation energy (E_a_^‡^)^66^, so that:

2 $$\alpha = \, - {\text{E}}_{{\text{a}}}^{\ddag } /{\text{R}}$$

α is the slope obtained from the calculation of 1/T vs. Ln [k_a_], and R is the gas constant (8.314 J/K mol)

3 $${\text{k}}_{{{\text{cat}}}} = \, ({\text{k}}_{{\text{B}}} /\hbar ) \times {\text{k}}_{{{\text{cat}}}}^{\ddag }$$

k_B_ and ℏ are the Boltzmann and Planck constants, respectively, and N is the Avogadro’s number.

4 $$\Delta {\text{G}}^{\ddag } = \, - {\text{RT }}\left[ {{\text{Ln k}}_{{{\text{cat}}}}^{\ddag } } \right]$$

T is the temperature in Kelvin.

The ∆G^‡^ is the changes in the Gibbs free energy of activation energy

5 $$\Delta {\text{H}}^{\ddag } = {\text{ E}}_{{\text{a}}}^{\ddag } - {\text{RT}}$$

The ∆H^‡^ is the changes in the enthalpy energy,

6 $$\Delta {\text{S}}^{\ddag } = \, \Delta {\text{H}}^{\ddag } - \Delta {\text{G}}^{\ddag } /{\text{T}}$$

The ∆S^‡^ is the changes in entropy energy.

[ΔG^‡^_Enzyme–Substrate_] is calculated as:

7 $$\Delta {\text{G}}^{\ddag }_{{{\text{Enzyme}} - {\text{Substrate}}}} = \, - {\text{RT }}\;{\text{Ln }}\;{\text{K}}_{{\text{i}}} \;{\text{that }}\;{\text{K}}_{{\text{i}}} = { 1}/{\text{K}}_{{\text{m}}}$$

Free energy of the transition state (TS) [ΔG^‡^_Enzyme–Substrate_] formation is obtained as:

8 $$\Delta {\text{G}}^{\ddag }_{{{\text{Enzyme}} - {\text{Transition state}}}} = \, - {\text{RT Ln }}\left( {{\text{k}}_{{{\text{cat}}}} /{\text{K}}_{{\text{m}}} } \right)$$

CelC307 temperature stability graph was used for calculation of irreversible inactivation, so the inactivation constant (k_in_ m^−1^) was calculated as:

9 $${\text{Ln }}\left( {\left[ {{\text{Act}}} \right]{\text{t}}/\left[ {{\text{Act}}} \right]0} \right) \, = \, - {\text{ k}}_{{{\text{in}}}} {\text{t}}$$

t is the enzyme incubation time, [Act]0 is the CelC307activity at time 0, and [Act]t is the activity at the time ‘t’ of reaction.

The CelC307 half-life (t_1/2_) was measured as:

10 $${\text{t}}_{{{1}/{2}}} = {\text{ ln2}}/{\text{k}}_{{{\text{in}}}} \left( {{\text{min}}} \right)^{{ - {1}}}$$

This goes on to calculate E_a_^#^ based on the equation of Arrhenius:

11 $${\text{k}}_{{{\text{in}}}} = {\text{ Ae}}\left( { - {\text{E}}_{{\text{a}}}^{\# } /{\text{RT}}} \right)$$

such that

12 $${\text{Ln}}\left[ {{\text{k}}_{{{\text{in}}}} } \right] \, = \, - {\text{E}}_{{\text{a}}}^{\# } /{\text{RT}}$$

and

13 $${\text{D }} = {\text{ RT}}/{\text{k}}_{{{\text{in}}}}$$

D is the decimal reduction time.

The values of ΔG^#^, ΔH^#^, and ΔS^#^ related to CelC307 were determined through applying Eqs. (7, 8, and 9) with some modification, including that in Eq. (11) E_a_^#^_in_ was replaced with E_a_.

### Kinetic analysis of the specific inhibitors’ mode of inhibition

The inhibition mechanism of the CelC307 enzyme was measured with two concentrations (0.5 and 1 mM) of three specific inhibitors with the highest inhibitory effects; fructose, lactose, and maltose. The kinetics of CelC307 activity in the inhibitors’ presence was determined by Michaelis–Menten plots using GraphPad Prism and Lineweaver–Burk plots using Excel 2019. Different concentrations of CMC were used as the enzyme’s substrate.

### Consent for publication

The authors have consent for publication.

## Supplementary Information

Supplementary Information.

## Data Availability

Data and material are available.
