# Supplementary material for: Expression, characterization, and activity optimization of a novel cellulase from the thermophilic bacteria Cohnella sp. A01
Source: Sci Rep. 2022 Jun 18;12:10301. doi: 10.1038/s41598-022-14651-7 (PMC9206686; doi:10.1038/s41598-022-14651-7)
Supplement: Supplementary file 1 — Supplementary Information. [file 41598_2022_14651_MOESM1_ESM.pdf]

Expression, characterization, and activity optimization of a novel cellulase from the thermophilic bacteria *Cohnella sp.* A01

Shima Mohammadi[1, †], Hossein Tarrahimofrad[1, †], Sareh Arjmand[2], Javad Zamani[1], Kamahldin Haghbeen[1], Saeed Aminzadeh[1]*

[1]Bioprocess engineering group, Institute of Industrial and Environmental Biotechnology, National Institute of Genetic Engineering and Biotechnology (NIGEB), Tehran, Iran

[2]Protein Research Center, Shahid Beheshti University, Tehran, Iran

***Corresponding Author; Saeed Aminzadeh**
E-mail: aminzade@nigeb.ac.ir

† Shima Mohammadi and Hossein Tarrahimofrad are joint first authors (equally contributed).

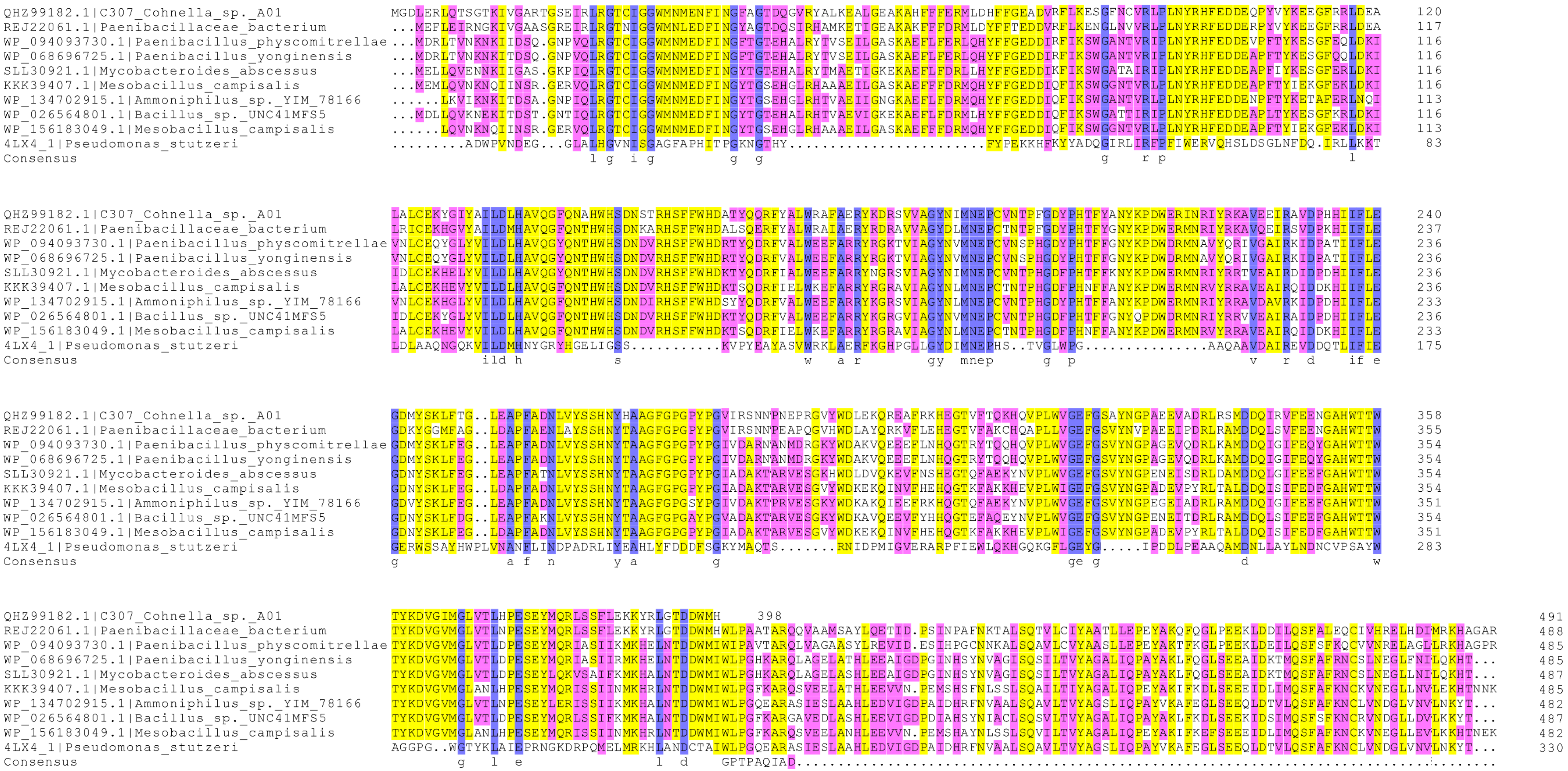

Figure S1. The results of multiple alignments of CelC307 amino acid sequence with some of the well-known cellulases (with the highest similarity) led to identifying the most conserved residues.

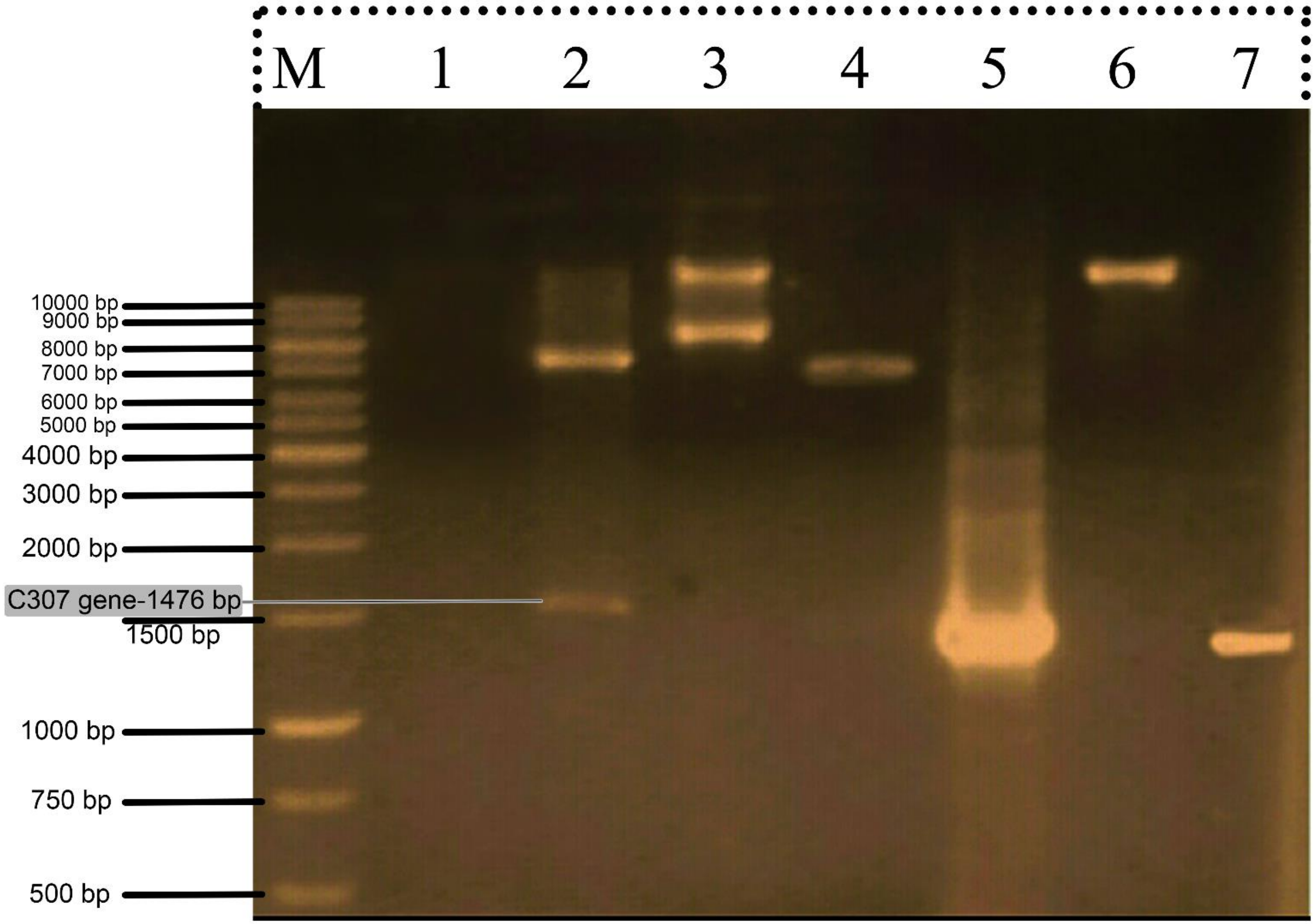

Figure S2. Analysis of cloning of recombinant CelC307. (A) M, DNA marker negative; 1, negative control; 2, Double digestion of recombinant pET26b(+) by *Xho*I and *Nde*I restriction enzyme; 3, empty pET26b(+) vector; 4, digested pET26b(+) vector; 5, PCR from genomic DNA of CelC307 sequence; 6, recombinant pET26b(+) containing CelC307 sequence; 7, PCR from recombinant pET26b(+) vector with specific primers for CelC307 sequence (colony PCR).

Table S1. Table of the Plackett-Burman design of seven factors.

Runs	Variables							Response (U/ml)
	pH	Temp. (˚C)	Shack (rpm)	Inoculum conc. (OD_{600})	Time (h)	Yeast extract (g/l)	Trypton (g/l)	
**1**	8	20	70	0.3	18	7.5	12.5	70
**2**	8	40	70	1	4	2.5	7.5	15
**3**	8	20	200	0.3	4	2.5	12.5	45.2
**4**	8	40	200	0.3	18	7.5	7.5	33.4
**5**	5	40	200	1	4	7.5	12.5	124.2
**6**	5	20	200	1	18	2.5	12.5	135
**7**	5	20	70	1	18	7.5	7.5	108.1
**8**	8	20	200	1	4	7.5	7.5	52.1
**9**	8	40	70	1	18	7.5	12.5	38
**10**	5	40	200	0.3	18	2.5	7.5	39.6
**11**	5	40	70	0.3	4	7.5	12.5	50.3
**12**	5	20	70	0.3	4	2.5	7.5	56.2

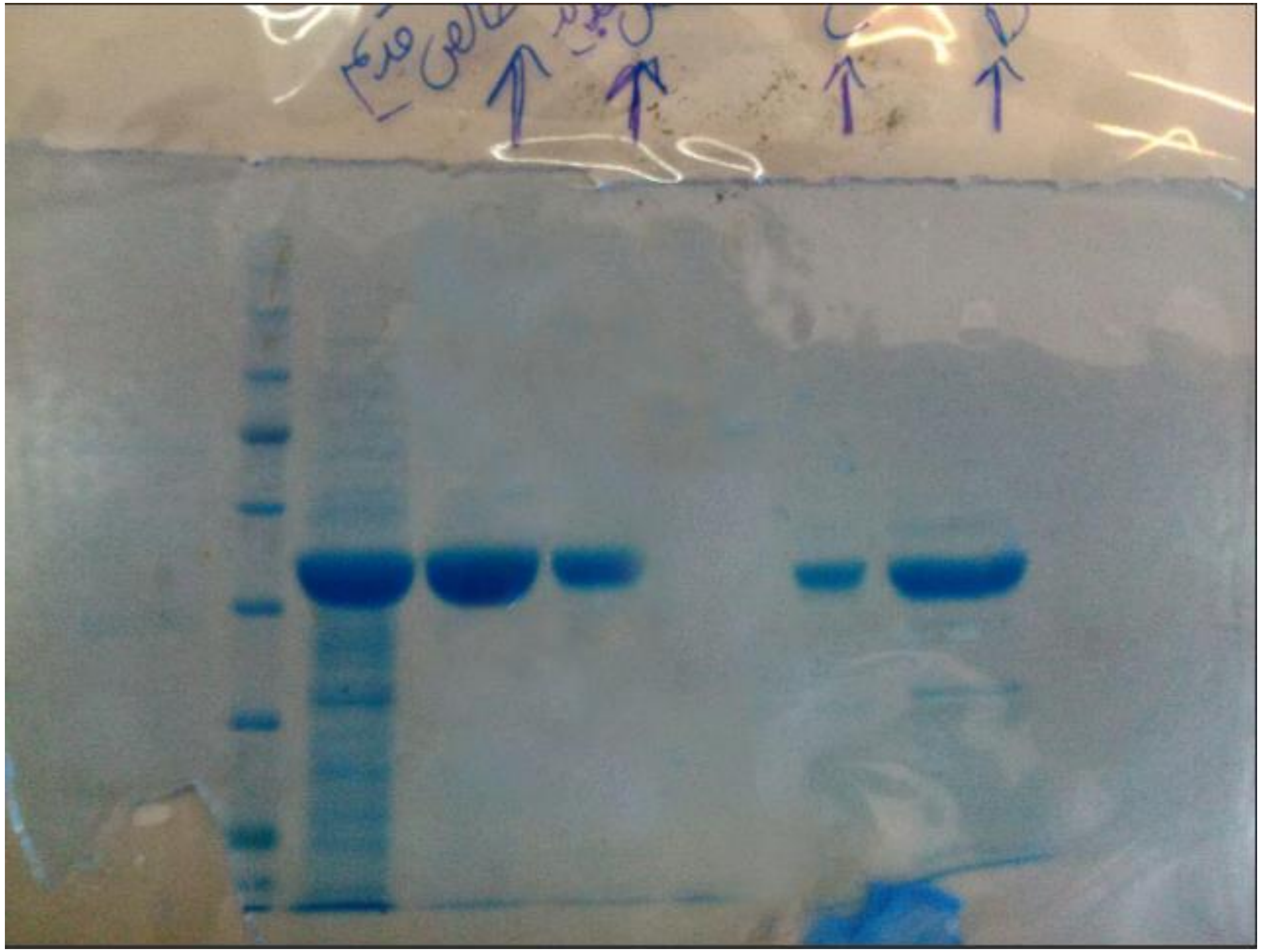

Figure S3. Full-length gel_Expression and Purification

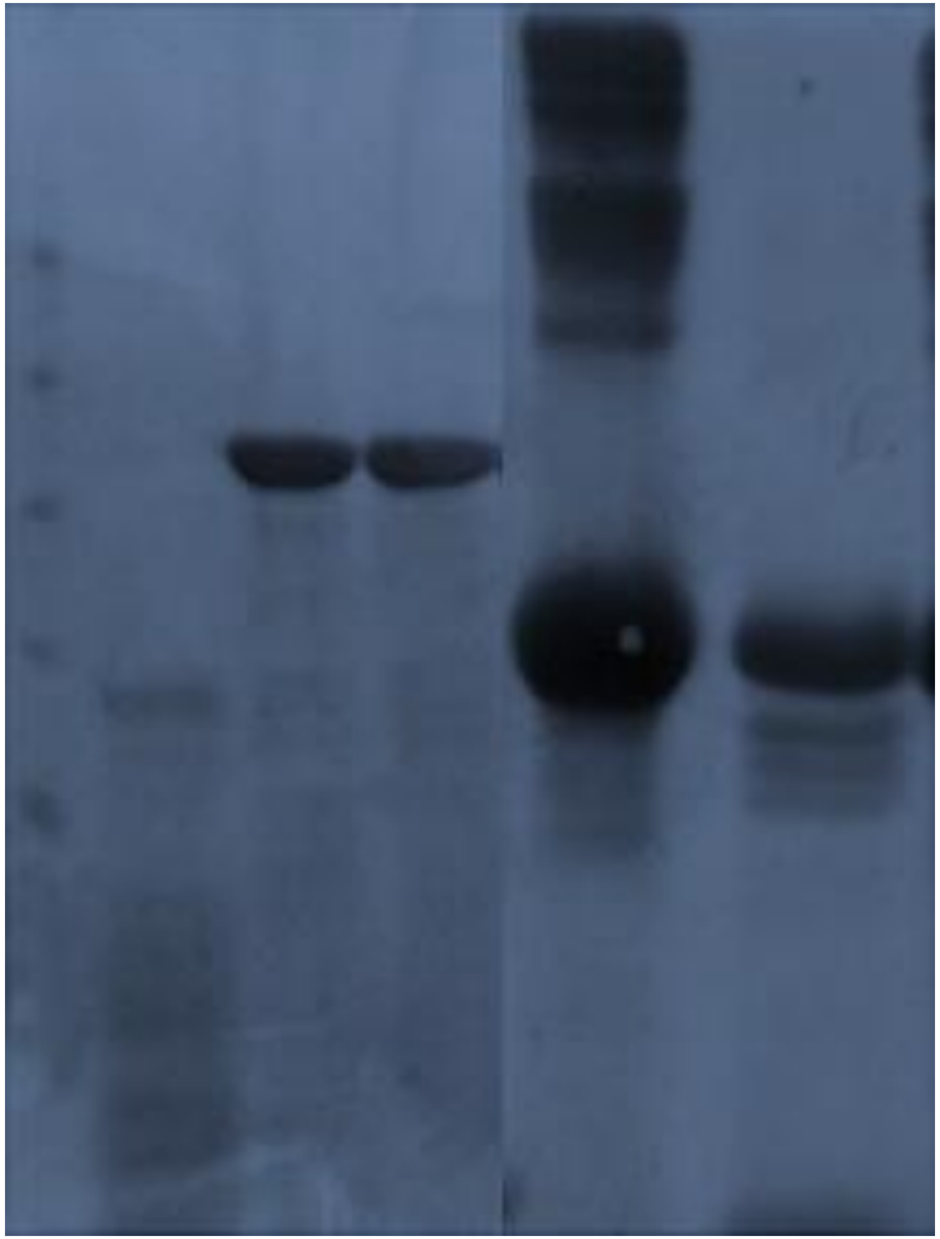

Figure S4. Full-length gel_Proteolytic digest resistance

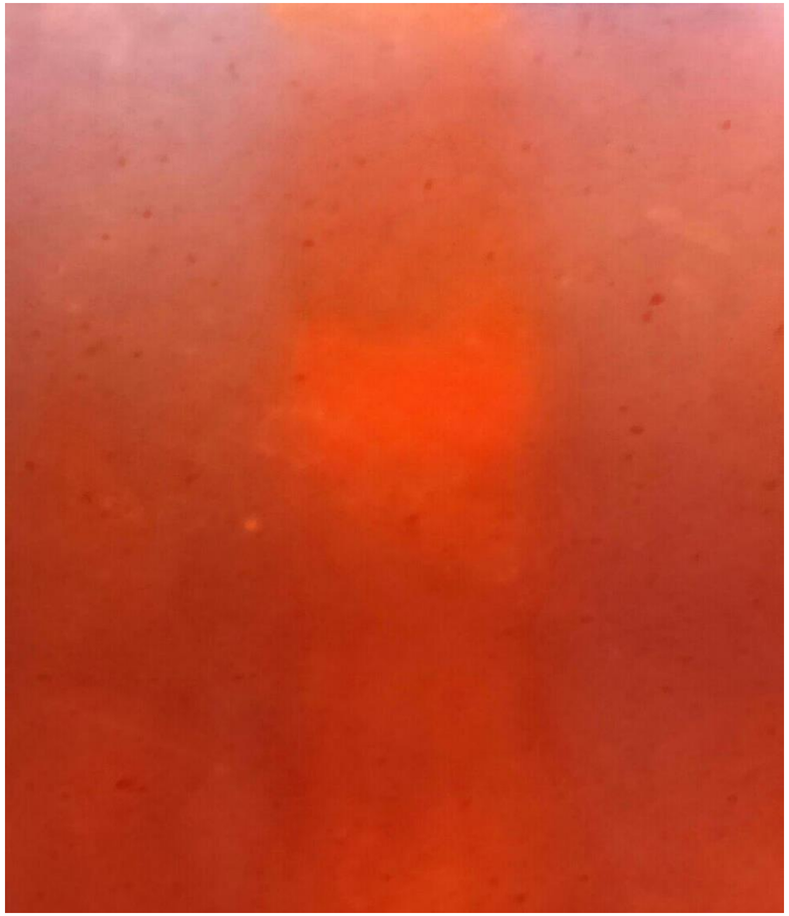

Figure S5. Full-length gel_Zymography

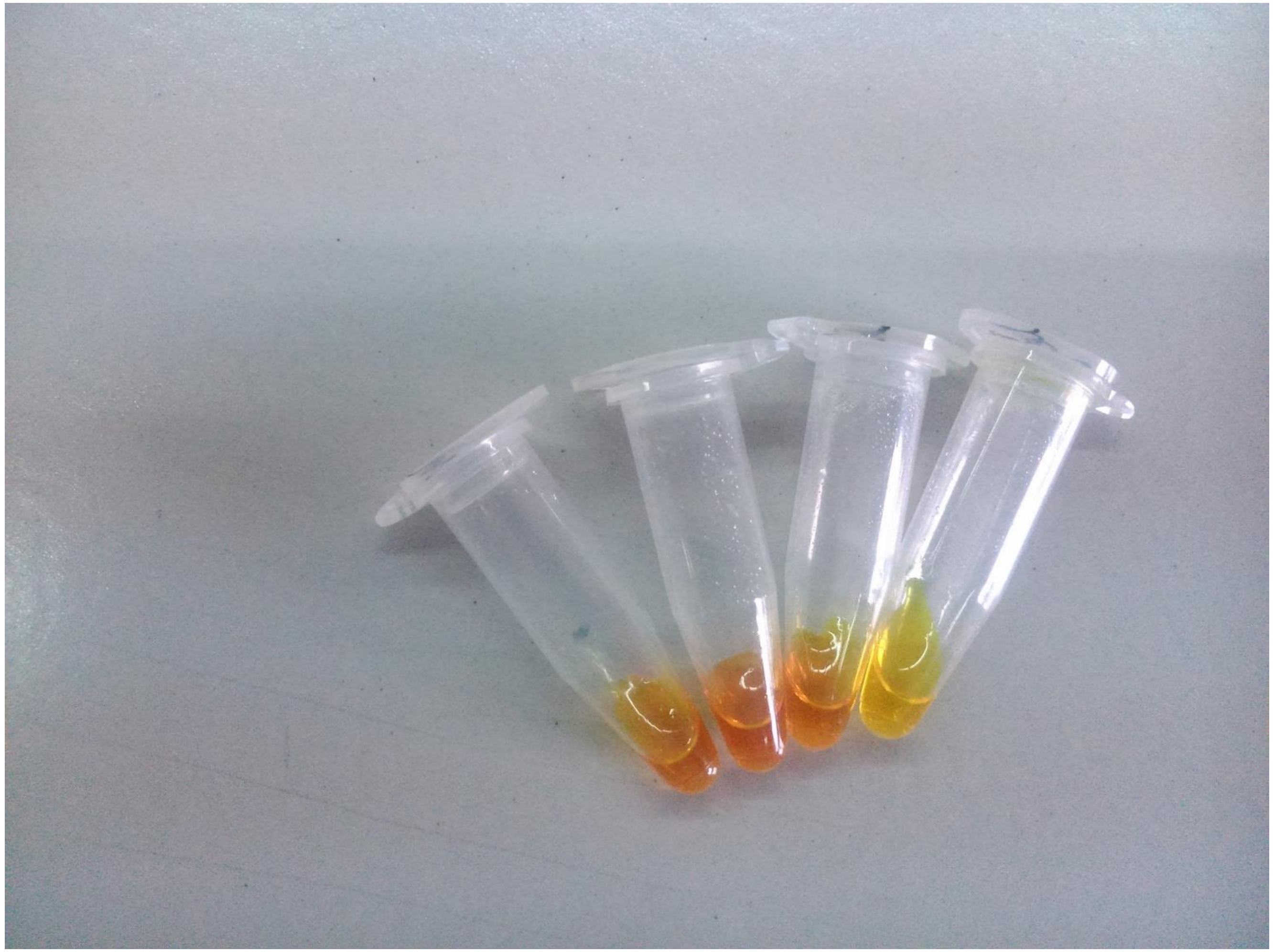

Figure S6. Full-length picture_Assay
